# Spatio‐Temporal Proteomic Landscape Reveals Early Warning Signals of Esophageal Squamous Cell Carcinoma Progression

**DOI:** 10.1002/advs.202514343

**Published:** 2026-01-08

**Authors:** Xumiao Li, Jie Yuan, Min Gao, Jibin Liu, Qinqin Wang, Yaqi Zhang, Mingtao Cao, Xiaolin Hu, Hui Yang, Jun Li, Chen Li, Xiaoguang Li, Hui Wang

**Affiliations:** ^1^ State Key Laboratory of Systems Medicine For Cancer, Center for Single‐Cell Omics School of Public Health Shanghai Jiao Tong University School of Medicine Shanghai China; ^2^ Institute of Oncology Affiliated Tumor Hospital of Nantong University Nantong China; ^3^ NHC Key Laboratory of Food Safety Risk Assessment China National Center for Food Safety Risk Assessment Beijing China; ^4^ Cancer Prevention and Treatment Office Yanting Cancer Hospital Mianyang China

**Keywords:** esophageal squamous cell carcinoma, dynamic network biomarker, GBP6, PARP1, spatio‐temporal proteomic landscape

## Abstract

Esophageal squamous cell carcinoma (ESCC) remains a lethal malignancy lacking reliable biomarkers for precancerous lesion monitoring and intervention. In this study, formalin‐fixed, paraffin‐embedded archived tissue biopsies from 134 individuals across two cohorts are collected, spanning five stages of ESCC progression. Laser capture microdissection is employed to isolate the epithelial lesion (L) and the adjacent non‐lesion (N) tissues. Proteomic profiling reveals a comprehensive spatio‐temporal landscape of ESCC progression, encompassing 4461 proteins. Dynamic network biomarker analysis indicates moderate dysplasia (MOD) as the critical turning stage, warranting clinical attention. A seven‐protein diagnostic panel (CCDC86, GBP6, PDCD6IP, C19orf53, SF3A3, GMPPB, ARPC5) with an area under the curve (AUC) of 0.956 achieves superior early detection, and the key signatures are validated by immunohistochemistry in an independent cohort. Functional validation using malignantly transformed HET‐1A cells, ESCC cells, and mouse xenograft models identifies GBP6 as a novel target: DNA damage‐induced progressive loss of GBP6 promotes ESCC progression by accelerating cell cycle and inducing epithelial‐mesenchymal transition. Critically, PARP1 inhibition rescues GBP6 loss by suppressing TP63 and prevents ESCC progression. Overall, this study provides a systematic proteomic atlas of ESCC progression, identifies MOD as a pivotal clinical decision point, and proposes PARP1‐TP63‐GBP6 axis targeting as a novel intervention strategy.

## Graphical abstract

This study constructs a proteomic atlas of esophageal squamous cell carcinoma (ESCC) progression using esophageal biopsy samples. It identifies moderate dysplasia as the critical turning stage and a seven‐protein panel for early detection. Functionally, GBP6 loss promotes ESCC progression via cell cycle and epithelial‐mesenchymal transition (EMT). Crucially, PARP1 inhibition could rescue GBP6 by suppressing TP63, proposing a novel intervention strategy.

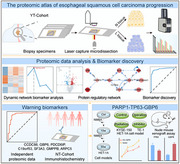

## 1 Introduction

Esophageal squamous cell carcinoma (ESCC) is the predominant type of esophageal cancer, a prevalent upper gastrointestinal malignancy worldwide [1, 2]. ESCC progresses from esophagitis (ESO) to mild dysplasia (MID), moderate dysplasia (MOD), severe dysplasia (SED), carcinoma in situ (CIS), and eventually invasive carcinoma [3]. Clinically, low‐grade intraepithelial neoplasia (LGIN, including MID and MOD) patients are usually followed without surgical treatment, whereas those with high‐grade intraepithelial neoplasia (HGIN, including SED and CIS) typically require treatment with endoscopic mucosal resection [3]. However, this treatment strategy is considered to be undertreated for LGIN and overtreated for HGIN, and thus scientific stratification systems are needed [3].

The prognosis for ESCC patients is dismal, but those diagnosed early fare better [4], highlighting the importance of early biomarkers. Exploring precancerous lesions provides a precious opportunity to reveal the evolutionary trajectories and biological mechanisms of cancer and to identify early biomarkers [5]. A few studies have focused on ESCC precancerous progression and found important molecules and mechanisms [6, 7, 8, 9, 10, 11], but comprehensive and cell‐type‐resolution proteomic studies are lacking. Unfortunately, accessing sufficient precancerous tissues for proteomic analysis remains challenging due to limited accessibility and size constraints in sample acquisition, especially in formalin‐fixed, paraffin‐embedded (FFPE) tissues containing cross‐linked proteins [12]. Additionally, biopsy samples primarily comprise normal epithelium, particularly in early lesions, with only minute areas representing true epithelial lesions. Recently, laser capture microdissection (LCM) based‐spatial proteomic methods, including our LCM‐MTA [13], allow the direct harvesting of specific cell subpopulations [14, 15], thereby drawing a precise proteomic atlas of true epithelial lesions from biopsies.

Here, we conducted a spatio‐temporal proteomic analysis of 112 LCM‐based epithelial lesion (L) and non‐lesion (N) tissues, covering the five key stages of ESCC progression. Our analysis delineated the protein profiles and dynamic changes associated with ESCC progression, with DNB analysis indicating MOD as the relatively benign‐malignant transition stage. Seven early warning biomarkers were identified by machine learning and validated by large‐scale immunohistochemistry (IHC). Further functional assays confirmed that the progressive loss of GBP6 promotes ESCC progression by accelerating cell cycle and inducing epithelial‐mesenchymal transition (EMT). We also identified PARP1 as an early target and demonstrated that its inhibition could rescue GBP6 loss by suppressing TP63. In summary, our study provides valuable insights for the early diagnosis, tracking, and treatment of ESCC.

## 2 Results

### 2.1 Spatio‐Temporal Proteomics Study Design for ESCC Progression

**FIGURE 1:**
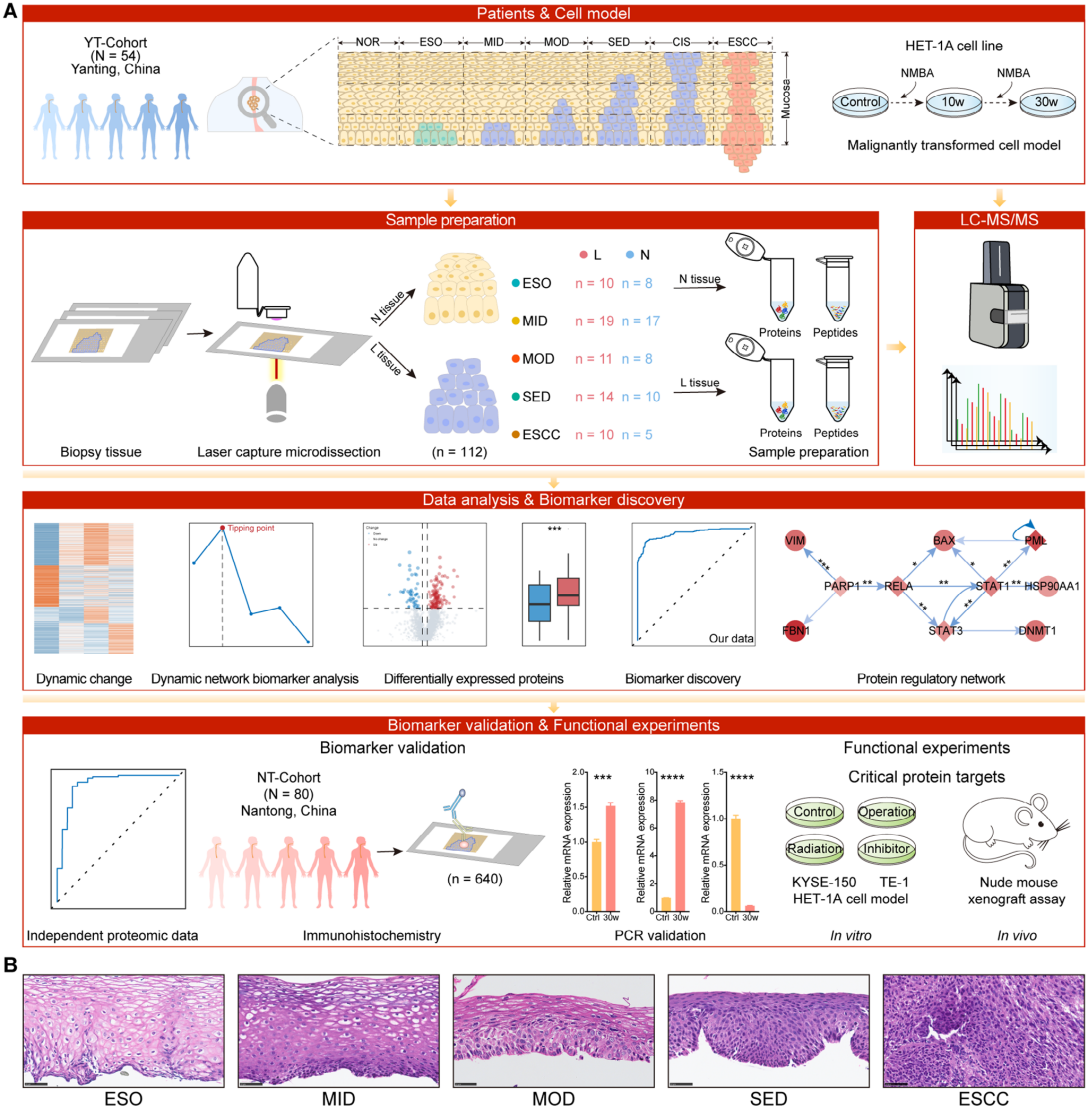
The workflow of protein profiling and early biomarker research for ESCC progression. (A) Scheme of the overall study design. (B) The hematoxylin and eosin (H&E) staining of the representative tissues during ESCC progression. Scale bars: 50 µm.

To investigate the molecular mechanisms underlying ESCC progression and to identify early warning biomarkers and therapeutic targets, we performed a spatio‐temporal proteomic analysis for ESO, MID, MOD, SED, and ESCC. Specifically, we obtained a total of 71 biopsy samples from 54 patients (YT‐Cohort, Table S1). Epithelial lesion tissue (ELT, L tissue) and adjacent non‐lesion tissue (ANT, N tissue) were precisely collected from biopsy samples by LCM, and finally, 10 ESO_L, 19 MID_L, 11 MOD_L, 14 SED_L, 10 ESCC_L, 8 ESO_N, 17 MID_N, 8 MOD_N, 10 SED_N, and 5 ESCC_N trace tissues were collected. We then performed Mass spectrometry (MS) analysis to shed light on the molecular mechanisms and early warning biomarkers of ESCC progression. In addition, we also collected 80 biopsy samples (NT‐Cohort), including 10 normal (NOR) esophageal epithelium, 11 ESO, 17 LGIN, 22 HGIN, and 20 ESCC tissues for immunohistochemistry (IHC) to validate biomarkers (Table S2). Finally, we established a malignant transformation model of human esophageal epithelial cells and performed functional experiments to validate critical molecular targets and investigate their biological mechanisms (Figure 1A,B).

### 2.2 Proteomic Landscape and Distinctions in L and N Tissues During ESCC Progression

**FIGURE 2:**
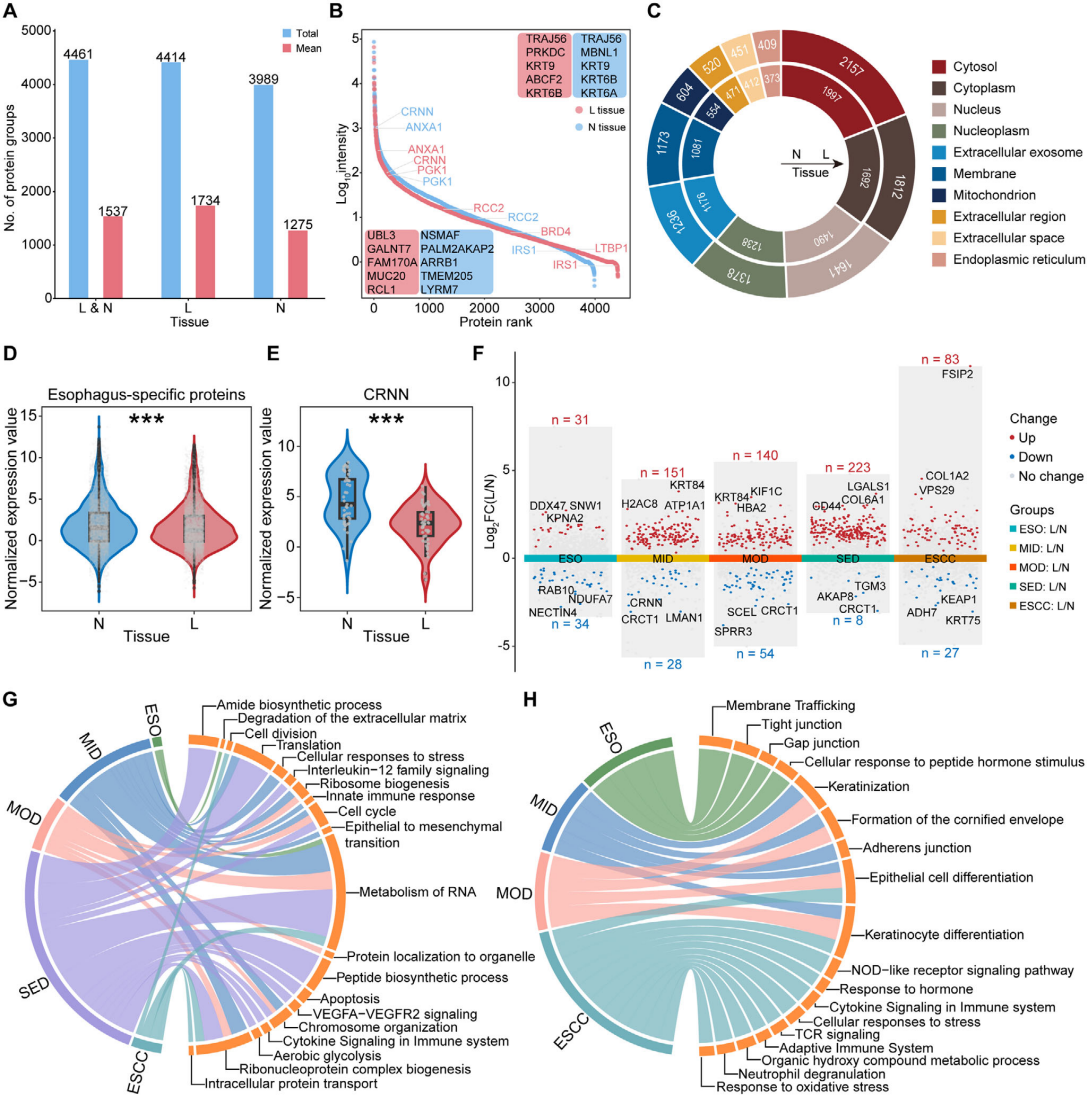
The protein profiles of ESCC progression and proteomic differences between L and N tissues. (A) Number of protein groups identified in all samples, L and N tissues, respectively. (B) The dynamic range of proteins identified in L (red) and N (blue) tissues. The top 5 high‐abundance (top) and top 5 low‐abundance (bottom) proteins are indicated in the box. (C) Doughnut chart shows the distribution of the top 10 subcellular compartments for identified proteins. (D,E) Boxplot shows the abundance of esophagus‐specific proteins (D) and CRNN (E) in L and N tissues. *p* value was calculated by the Wilcoxon rank‐sum test (^***^ *p* <0.001). (F) The plot displays the differentially expressed proteins (DEPs) between N and L tissues across all stages. (G,H) The chord diagram shows the representative pathways of upregulated (G) and downregulated (H) proteins across various stages.

Using data‐independent acquisition (DIA) proteomics, we identified a total of 4461 protein groups, including 4414 in L tissues and 3989 in N tissues (Figure 2A). On average, we identified 1734 and 1275 protein groups in L and N tissues, respectively, with significantly more proteins identified in L tissues (Figure 2A; Figure S1A,B). The intensity of quantified proteins spanned five orders of magnitude, and some biomarkers were identified in both high and low abundance ranges, demonstrating the unbiased and reliable data (Figure 2B). The proteins were mainly distributed in the cytoplasm, nucleus, and mitochondria, consistent with a previous study (Figure 2C) [16]. Interestingly, L tissues contained more cancer‐related proteins and drug targets than N tissues (Figure S1C; Table S3). In L tissues, 3366 proteins were common across all stages, while 2430 proteins were identified across all stages in N tissues (Figure S1D,E). Additionally, 310 proteins were commonly identified in L tissue from precancerous lesions and ESCC. These proteins are involved in EMT and glycolysis, suggesting that oncogenic events may be present at the precancerous stage.

Partial Least Squares Discriminant Analysis (PLSDA) revealed significant differences in protein profiles between L and N tissues (Figure S1F), highlighting the heterogeneity of bulk samples and the marked proteomic distinctions between the two tissues. We also conducted separate PLSDA on L and N tissues, aligning each with its respective pathological stages, revealing distinct protein expression profiles (Figure S1G,H). In L tissues, MID exhibited greater similarity to ESO than MOD, while MOD was closer to SED and ESCC. These findings suggest that individuals with MOD exhibit a protein background more similar to SED and ESCC.

To compare the proteomic differences between L and N tissues, we first investigated the overlap of proteins at each stage and found that approximately 98% of the proteins in N tissues were concurrently identified in L tissues (Figure S2A). We then examined the changes in abundance patterns of shared proteins, and found that proteins varied greatly between L and N tissues, suggesting their potential biological functions in promoting ESCC progression (Figure S2B). Furthermore, we identified 151 and 155 esophagus‐specific proteins in L and N tissues, respectively, and these proteins, such as CRNN, KRT4, TGM1, and KLK10 were significantly downregulated in L tissues (Figure 2D,E; Figure S2C–E), indicating a significant loss of normal esophageal properties.

We then sought to identify differentially expressed proteins (DEPs) between L and N tissues (Figure 2F–H; Table S4). At ESO, upregulated proteins were involved in RNA metabolism and extracellular matrix degradation, whereas downregulated proteins were enriched in tight junction and gap junction, indicating potential damage to the epithelium. At MID and MOD, upregulated proteins were involved in RNA metabolism, cell cycle, and EMT, whereas downregulated proteins were enriched in keratinization, formation of the cornified envelope, and epithelial cell differentiation. These findings suggest that oncogenic‐related pathways are activated in L tissues, while proteins involved in the primary functions of the esophagus are lost, indicating that tumorigenesis may occur during the MID and MOD, which is similar to that reported in previous studies [8, 17]. Among 110 DEPs at ESCC, 83 upregulated proteins were enriched in RNA metabolism and cell division, while 27 downregulated proteins were involved in cytokine signaling and the adaptive immune system. We also annotated 45 esophagus‐specific proteins in DEPs and identified LCN1 as a potential biomarker (Figure S2F,G). Collectively, we delineated the protein profiles of ESCC progression in temporal and spatial dimensions and found significant differences between L and N tissues.

### 2.3 Protein Dynamic Changes and the Tipping Stage of ESCC Progression

**FIGURE 3:**
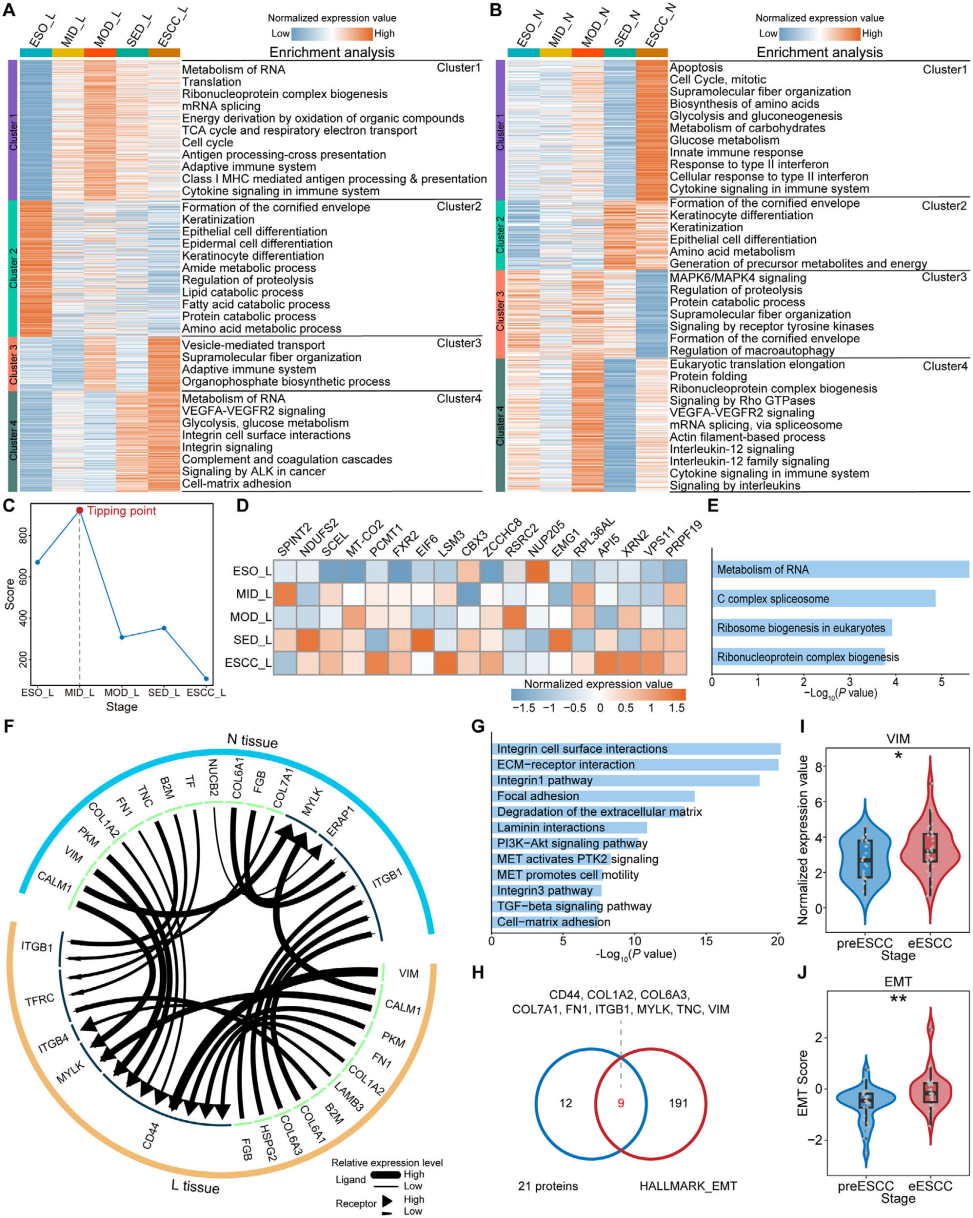
Protein dynamic changes and the tipping stage of ESCC progression. (A,B) The protein dynamic changes of L (A) and N tissues (B) were divided into four clusters by Mfuzz. (C) The composite index (CI) scores from DNB analysis in L tissues. (D,E) The expression levels (D) and enrichment analysis (E) of DNB proteins in L tissues. (F) The interaction network based on receptor‐ligand pairs between L and N tissues at the MOD stage was constructed using iTALK. (G) The enrichment analysis of proteins that are involved in the interaction network (F). (H) A Venn diagram shows the overlap between the proteins involved in the interaction network (F) and the EMT‐related proteins. (I,J) Comparison of VIM (I) and EMT scores (J) between preESCC and eESCC phases. *p* value was calculated by Student's t test (^*^ *p* <0.05, ^**^ *p* <0.01).

We systematically analyzed the protein dynamic changes during ESCC progression to reveal biological events associated with tumorigenesis in a temporal dimension. In L tissues, we obtained four protein clusters by Mfuzz clustering (Figure 3A; Table S5). Cluster 1 proteins exhibited an overall increasing trend and reached the highest expression at MOD, and were enriched in tumorigenesis‐related pathways, metabolism, and immune‐related pathways. This suggests that tumorigenic events may occur at the MOD. Cluster 2 proteins were progressively downregulated and were involved in the primary functions of the esophagus such as keratinization, epithelial cell differentiation, and metabolic processes, indicating a significant loss of normal esophageal function. Interestingly, cluster 3 proteins showed fluctuations, with higher expression at MOD and ESCC, and were enriched in vesicle‐mediated transport and supramolecular fiber organization. Cluster 4 proteins are gradually upregulated, peaking at ESCC, with enrichment in RNA metabolism, glycolysis, and integrin signaling. Notably, integrin signaling is strongly associated with tumor initiation, progression, and metastasis, and exerts its apparent effect in the EMT [18]. Furthermore, we also performed Mfuzz clustering on N tissues (Figure 3B; Table S5). Cluster 2 proteins showed a tendency to gradually upregulate expression, and were involved in keratinization, epithelial cell differentiation, and amino acid metabolism, which is opposite to the expression trend of Cluster 2 proteins in L tissues, suggesting that there may be a compensatory mechanism. Cluster 4 proteins showed fluctuating changes and peaked at MOD, and were mainly involved in translation and immune‐related pathways, suggesting that the MOD may be a critical stage. In summary, the protein dynamics of in situ tissues suggest that ESCC progression is not gradual and monotonic, but rather a complex process with non‐linear and drastic transitions [19, 20].

Furthermore, a scientifically sound stratification system is needed to assess the risk of progression in patients with precancerous lesions [3]. As ESCC progression is a complex, non‐linear transformation process, we can apply DNB analysis to discern the tipping stage by investigating the dynamic and network aspects of proteomic data during ESCC progression [20]. This implies that we can identify the tipping stage in ESCC progression, consequently identifying the precancerous phase of ESCC (preESCC phase) and the early phase of ESCC (eESCC phase). In L tissues, DNB analysis identified MID as the tipping stage and yielded 18 DNB proteins involved in RNA metabolism and ribosome biogenesis (Figure 3C–E). According to the DNB theory, the precancerous lesions enter a relatively irreversible state after passing through the MID, i.e., the MOD marks the commencement of the relatively malignant and irreversible state [19, 20]. Thus, we combined the ESO and MID into the preESCC phase and the MOD, SED, and ESCC into the eESCC phase. In parallel, DNB analysis of N tissues identified MOD as the tipping stage and obtained 19 DNB proteins, mainly enriched in epithelial cell migration (Figure S3A–C). These findings also preliminarily suggest that MOD represents a critical stage in the transition from the relatively benign to malignant state during ESCC progression, which highly merits further validation through longitudinal cohorts in future studies. Further analysis identified stage‐specific proteins, with MOD having the highest number of stage‐specific proteins, highlighting its unique protein pattern and critical role in ESCC progression (Figure S3D,E).

We then performed the iTALK analysis of L and N tissues at MOD, a method for characterizing and illustrating the ligand‐receptor mediated intercellular cross‐talk signals in multicellular systems [21]. The interaction network of potential receptor‐ligand pairs between L and N tissues revealed a clear interaction that underscores the role of N tissues in driving ESCC progression (Figure 3F). For instance, we found that B2M was gradually upregulated in N tissues, whereas its expression fluctuated in L tissues (Figure S3F). Notably, *B2M* was significantly associated with patient prognosis (Figure S3G), and a previous study reported that mesenchymal stromal cell‐derived B2M promoted the EMT in ESCC cells [22]. The proteins involved in the network were enriched in integrin signaling, ECM‐receptor interaction, PI3K‐Akt signaling, and TGF‐β signaling pathway (Figure 3G). Fascinatingly, the majority of these pathways intricately intertwine with the EMT [23]. We further found that 9 proteins were annotated as EMT‐related proteins (Figure 3H). Additionally, a comparative analysis of VIM and EMT scores between preESCC and eESCC phases unveiled the pronounced activation of EMT (Figure 3I,J; Figure S3H). In summary, the foregoing findings strongly indicate that the MOD serves as a relatively benign‐malignant transition stage during ESCC progression, with the EMT likely playing a pivotal role.

### 2.4 The Proteomic Differences Between preESCC and eESCC Phases

**FIGURE 4:**
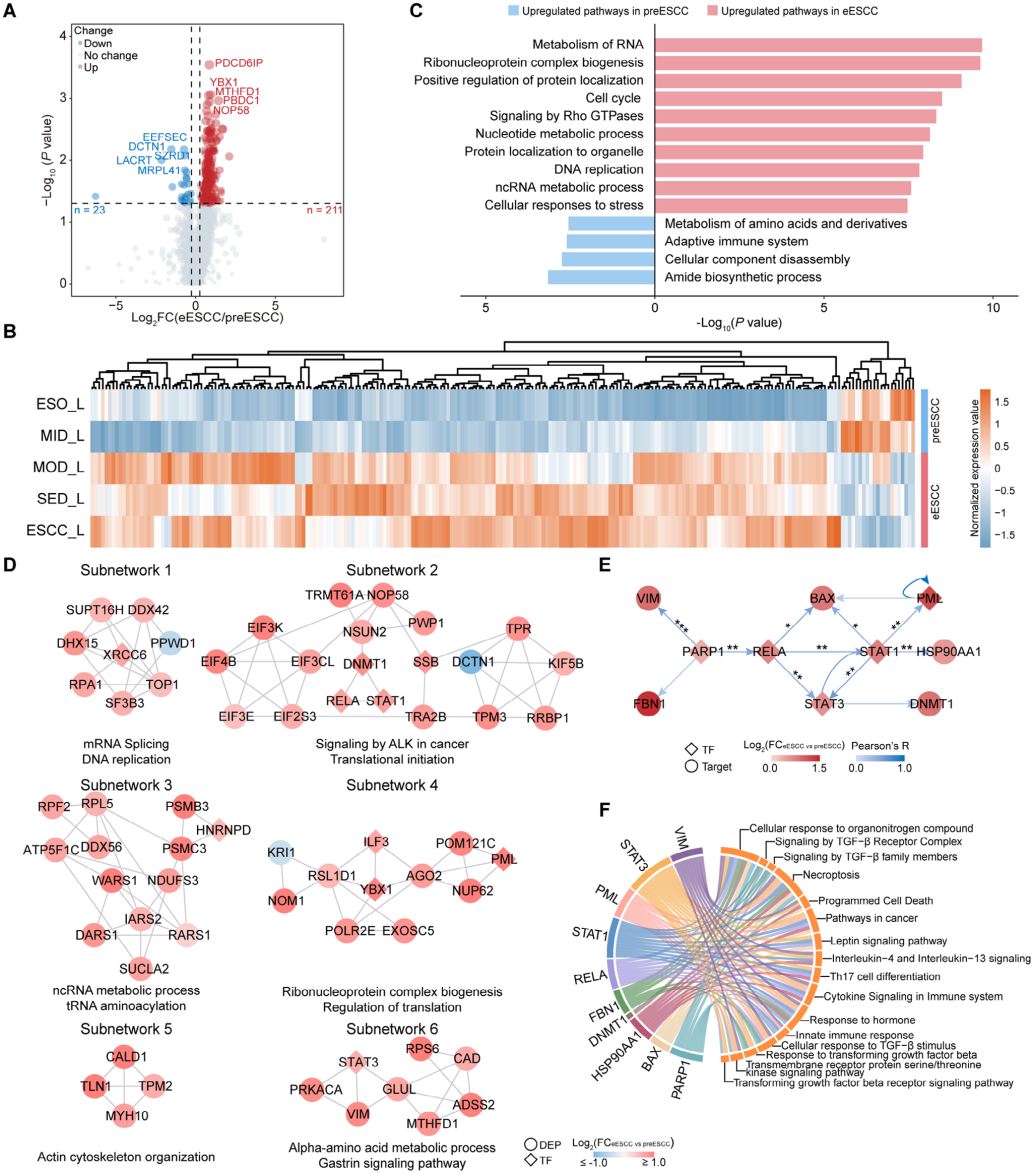
The proteomic differences between preESCC and eESCC phases. (A) Volcano plot showing the DEPs between preESCC and eESCC phases in L tissues. (B) The expression levels of 234 DEPs (A) across various stages in L tissues. (C) The enrichment analysis of 234 DEPs (A). (D) The functional subnetworks are extracted from the PPI network of DEPs. (E) The core transcriptional regulatory network was constructed based on TF‐target protein pairs. *p* value was calculated by Pearson's correlation analysis (^*^ *p* <0.05, ^**^ *p* <0.01, ^***^ *p* <0.001). (F) The chord diagram shows the pathways of ten proteins involved in the transcriptional regulatory network (E).

A total of 234 DEPs were found in L tissues, with 211 proteins significantly upregulated and 23 downregulated in the eESCC phase (Figure 4A,B; Table S6). The upregulated proteins were enriched in RNA metabolism and ribonucleoprotein complex biogenesis, while the downregulated proteins were involved in amino acid metabolism and the adaptive immune system (Figure 4C; Table S7). We first constructed a protein‐protein interaction (PPI) network of DEPs and DNB proteins, primarily involved in RNA metabolism and ribonucleoprotein complex biogenesis, aligning with the function of DNB proteins (Figure S4A–C; Table S8). In addition, we extracted the molecules in the ESCC development region from Chen et al. [11] and had 8 overlaps with our DEPs (Figure S4D). Consistent with previous findings [11], these EDR proteins showed a trend towards overall upregulated expression, particularly as early as MOD.

We also constructed a PPI network comprising 204 DEPs, of which 19 DEPs were annotated as transcription factors (TFs) (Figure S4E; Table S9). Subsequently, six functional subnetworks were extracted, revealing the key protein regulatory networks and hub proteins involved in ESCC progression (Figure 4D). We also found that the mRNA expression of SUPT16H, TOP1, SUCLA2, KRI1, CALD1, and MTHFD1 within these subnetworks were significantly associated with patient prognosis (Figure S4F–K).

Intriguingly, we noticed that DEPs annotated as TFs were extensively involved in forming subnetworks, suggesting that TFs and associated regulatory networks may play a critical role in promoting ESCC progression. Therefore, we obtained a core transcriptional regulatory network based on TF‐target protein pairs (Figure 4E; Figure S5A,B). The TFs and targets were significantly upregulated and involved in TGF‐β signaling and immune‐related pathways (Figure 4F). Notably, VIM and FBN1 are associated with EMT, and TGF‐β signaling also plays a central role in EMT induction [23]. We also found that Poly(ADP‐ribose) polymerase 1 (PARP1) is upstream of the entire network to regulate other targets. PARP1 is a well‐recognized molecular target closely associated with DNA repair, chromatin remodeling, and cell division [24]. PARP1 was also associated with VIM and FBN1 at both mRNA and protein levels and showed upregulated expression in tumor tissue (Figure S5A–D). These results suggest that PARP1, which is closely related to DNA repair and EMT, could be a promising target for early intervention. Finally, we identified DEPs between preESCC and eESCC phases in N tissues and constructed PPI networks for both DEPs with DNB proteins and DEPs alone, extracting key functional subnetworks. These findings provide unique insights into the mechanisms underlying ESCC progression (Figure S6A–F, Tables S10–S13).

### 2.5 Identifying Early Warning Biomarkers for ESCC Progression

**FIGURE 5:**
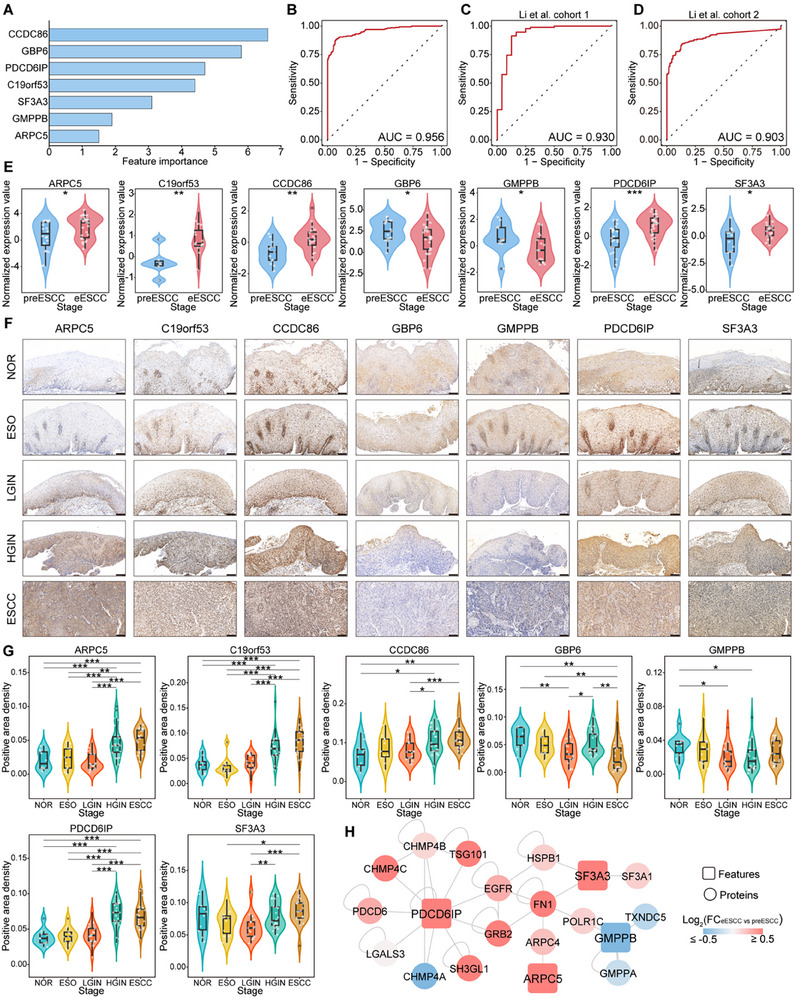
Identifying early warning biomarkers of ESCC by machine learning. (A) The importance rank of the seven most informative features selected by SVM‐RFE. (B) ROC curve of the seven most informative features to discriminate eESCC from preESCC in our data. (C,D) ROC curve of selected features to discriminate ESCC from normal tissue in Li et al. cohort 1 (C) and Li et al. cohort 2 (D). (E) Violin plots show the expression of seven features in preESCC and eESCC phases. *p* value was calculated by Student's t‐test or Wilcoxon rank‐sum test. (F) Representative images show immunohistochemistry staining of biomarkers at different stages. Scale bar, 100 µm. (G) Comparison of the positive area density of seven tissue biomarkers at different stages. *p* value was calculated by Student's t‐test. (H) The PPI network between the features and other proteins was constructed using the Human Reference Interactome (HuRI) database. ^*^ *p* <0.05, ^**^ *p* <0.01, ^***^ *p* <0.001.

We aimed to develop a machine learning model to identify early biomarkers and evaluate the ability of a protein panel to distinguish eESCC from preESCC. We first benchmarked 10 machine learning algorithms and selected the support vector machine (SVM) for its superior performance (Figure S7A,B; Table S14). Using the SVM‐recursive feature elimination (SVM‐RFE) algorithm, we initially selected 61 features, achieving an AUC of 0.917 (Figure S7C,D). Among these features, three proteins associated with the EMT, including TPM2, VIM, and CALD1, were significantly upregulated in the eESCC phase (Figure S7E,F). We further shrank the number of features and identified the seven most informative features, including CCDC86, GBP6, PDCD6IP, C19orf53, SF3A3, GMPPB, and ARPC5 (Figure 5A; Figure S7G). Subsequently, we constructed an SVM model and achieved good performance (AUC = 0.956) in distinguishing eESCC from preESCC (Figure 5B). We partially validated the model using published proteomic data of normal and ESCC samples from two independent cohorts [17, 25], and the model maintained good performance (Figure 5C,D). Taken together, this protein panel demonstrates effectiveness in distinguishing early ESCC patients and warrants further validation in longitudinal studies to expedite clinical translation.

We then compared the expression pattern of seven features and found that GBP6 and GMPPB were downregulated, while the other five features were upregulated in the eESCC phase (Figure 5E). Given the widespread use and low cost of IHC in clinical practice, we performed IHC staining of these features to validate early warning biomarkers for diagnosing early ESCC. IHC staining of tissues from an independent NT‐Cohort yielded similar results, further demonstrating the clinical value of these early warning biomarkers (Figure 5F,G). Finally, we further explored proteins interacting with these informative features and constructed a PPI network involving proteins enriched in cell migration and motility, EMT, and PI3K‐Akt‐mTOR signaling, aligning with our results above and emphasizing the key role of EMT (Figure 5H; Figure S7H). Collectively, these results provide reliable early warning biomarkers and potential intervention targets for ESCC.

### 2.6 Progressive Loss of GBP6 Promotes ESCC Progression by Accelerating the Cell Cycle and Inducing EMT

We next investigated the potential of these warning biomarkers as early intervention targets for ESCC. Based on feature importance ranking, the top three early‐warning biomarkers were the proteins CCDC86, GBP6, and PDCD6IP (Figure 5A). Among these early warning biomarkers, guanylate binding protein family member 6 (GBP6) was the only esophagus‐specific protein found in the Human Protein Atlas (HPA, https://www.proteinatlas.org/) database and was found to be particularly enriched in suprabasal keratinocytes, squamous epithelial cells, and basal keratinocytes [26]. These keratinocytes and squamous epithelial cells constitute the fundamental building blocks of normal esophageal squamous epithelium. The pathogenesis of ESCC is fundamentally driven by the dysregulation of the intrinsic programs that regulate the stratification and differentiation of these cells [27]. However, the role of GBP6 in the development and progression of ESCC remains elusive. More importantly, the underlying mechanism responsible for its progressive loss and the potential clinical implications are still unknown. This suggests that the GBP6 may be a novel and promising target for early ESCC that warrants further functional investigation.

**FIGURE 6:**
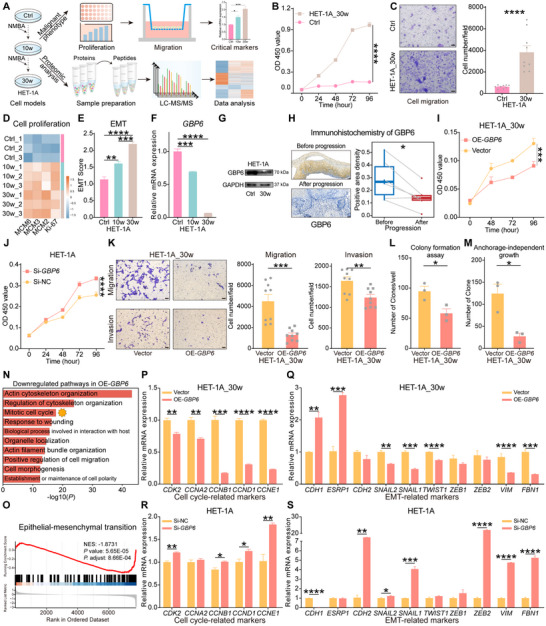
Progressive loss of GBP6 drives ESCC development by accelerating cell cycle and inducing EMT. (A) Schematic diagram of establishing and validating the HET‐1A cell model. (B) Comparison of cell proliferation between HET‐1A and HET‐1A_30w cells. (C) Representative images (left) and statistics (right) showing the comparison of migration between HET‐1A and HET‐1A_30w cells. Scale bar, 50 µm. (D) Protein expression levels of representative cell proliferation markers in the HET‐1A model. (E) Comparison of EMT scores in HET‐1A model. (F,G) Comparison of the mRNA (F) and protein (G) expression of GBP6 in the HET‐1A model. (H) Representative images (left) and quantitative statistics (right) of immunohistochemistry staining for GBP6 before and after progression. Scale bar, 50 µm. (I) Effects of *GBP6* overexpression on the proliferation of HET‐1A_30w cells. (J) Effects of *GBP6* knockdown on the proliferation of HET‐1A cells. (K) Representative images (left) and statistics (right) show the effects of *GBP6* overexpression on HET‐1A_30w cell migration and invasion. Scale bar, 50 µm. (L,M) Effects of *GBP6* overexpression on HET‐1A_30w cell colony formation (L), and anchorage‐independent growth (M). (N) The significantly downregulated pathways after *GBP6* overexpression in HET‐1A_30w cells. (O) Gene Set Enrichment Analysis (GSEA) demonstrated significant changes in the EMT process upon *GBP6* overexpression in HET‐1A_30w cells. (P–S) Quantitative PCR shows the effects of *GBP6* overexpression (P,Q) and knockdown (R,S) on the altered expression of genes involved in cell cycle (P,R) and EMT (Q,S) in HET‐1A_30w and HET‐1A cells. The *p* values were calculated by unpaired Student's t‐test (C, E, K‐M, P‐S), paired Student's t‐test (H), or Dunnett's test (B, F, I, and J). ^*^ *p* <0.05, ^**^ *p* <0.01, ^***^ *p* <0.001, ^****^ *p* <0.0001.

To explore the potential of GBP6 as an early ESCC target and its underlying mechanisms, we first used N‐nitrosomethylbenzylamine (NMBA) to induce malignant transformation in normal human esophageal epithelial cells HET‐1A, and thus established a malignantly transformed HET‐1A model (Figure 6A). After induction, cell proliferation and migration abilities were significantly enhanced (Figure 6B,C). We then performed the proteomic analysis of the HET‐1A model, revealing distinct differences in its protein expression profiles and significant upregulation of cell proliferation markers (Figure 6D; Figure S8A,B). Consistent with our previous results, EMT was also significantly activated in the HET‐1A model (Figure 6E; Figure S8C,D). Meanwhile, the dynamic changes in proteins during the malignant transformation of HET‐1A cells were similar to those in L tissue, such as significant upregulation of proteins related to the cell cycle, DNA repair, and RNA metabolism, and significant downregulation of proteins related to metabolism (Figure S8E). Consistent with the Transwell assay, proteins associated with the positive regulation of cell migration also showed significantly upregulated expression (Figure S8E,F). Additionally, we also verified the mRNA expression of seven biomarkers in the HET‐1A model (Figure 6F; Figure S8G). In conclusion, the results indicate that the HET‐1A cell model accurately reflects the biological processes of early ESCC progression.

GBP6 was significantly downregulated in the HET‐1A model, a finding that was also confirmed in the TCGA cohort and a previous study (Figure 6F,G; Figure S9A,B) [28]. We further validated GBP6 using longitudinal tissue samples from patients who progressed from preESCC to eESCC, and the results demonstrated a significantly reduced expression level of GBP6 after progression (Figure 6H). We then performed gene operation in HET‐1A_30w cells, human ESCC TE‐1 cells, and normal human esophageal epithelial cells HET‐1A to explore the effect of *GBP6* on malignant cell phenotypes (Figure S9C). The results showed that *GBP6* overexpression significantly suppressed the proliferation, migration, and invasion of HET‐1A_30w and TE‐1 cells, and *GBP6* knockdown significantly promoted the proliferation and migration of HET‐1A cells (Figure 6I–K; Figure S9D–G). Crucially, we found that *GBP6* overexpression significantly reduced the tumorigenicity of HET‐1A_30w and TE‐1 cells, as determined by colony formation and anchorage‐independent growth assays (Figure 6L,M; Figure S9H,I).

To further investigate the downstream mechanisms of GBP6, we performed proteomic analysis on the control and *GBP6*‐overexpressing groups of HET‐1A_30w cells (Figure S9J,K). By identifying DEPs and conducting functional enrichment analysis, we discovered that *GBP6* overexpression resulted in significant downregulation of pathways related to cell cycle and cell migration, while pathways related to metabolism were upregulated (Figure 6N; Figure S9L). Gene set enrichment analysis (GSEA) also demonstrated significant suppression of the EMT process (Figure 6O; Figure S9M). Based on these findings, we hypothesize that the malignant phenotypes induced by GBP6 in ESCC may be closely associated with the regulation of cell cycle and EMT processes. Ultimately, we found that *GBP6* overexpression significantly decreased the expression levels of cell cycle‐related markers and inhibited the EMT process in HET‐1A_30w and TE‐1 cells (Figure 6P,Q; Figure S9N,O). Conversely, *GBP6* knockdown significantly promoted the cell cycle and EMT in HET‐1A cells (Figure 6R,S). In summary, these results suggest that esophagus‐specific protein GBP6 is a novel ESCC biomarker, and that progressive loss of GBP6 could promote ESCC progression by accelerating the cell cycle and inducing EMT.

### 2.7 PARP1 Inhibition Rescues DNA Damage‐Induced Progressive Loss of GBP6

**FIGURE 7:**
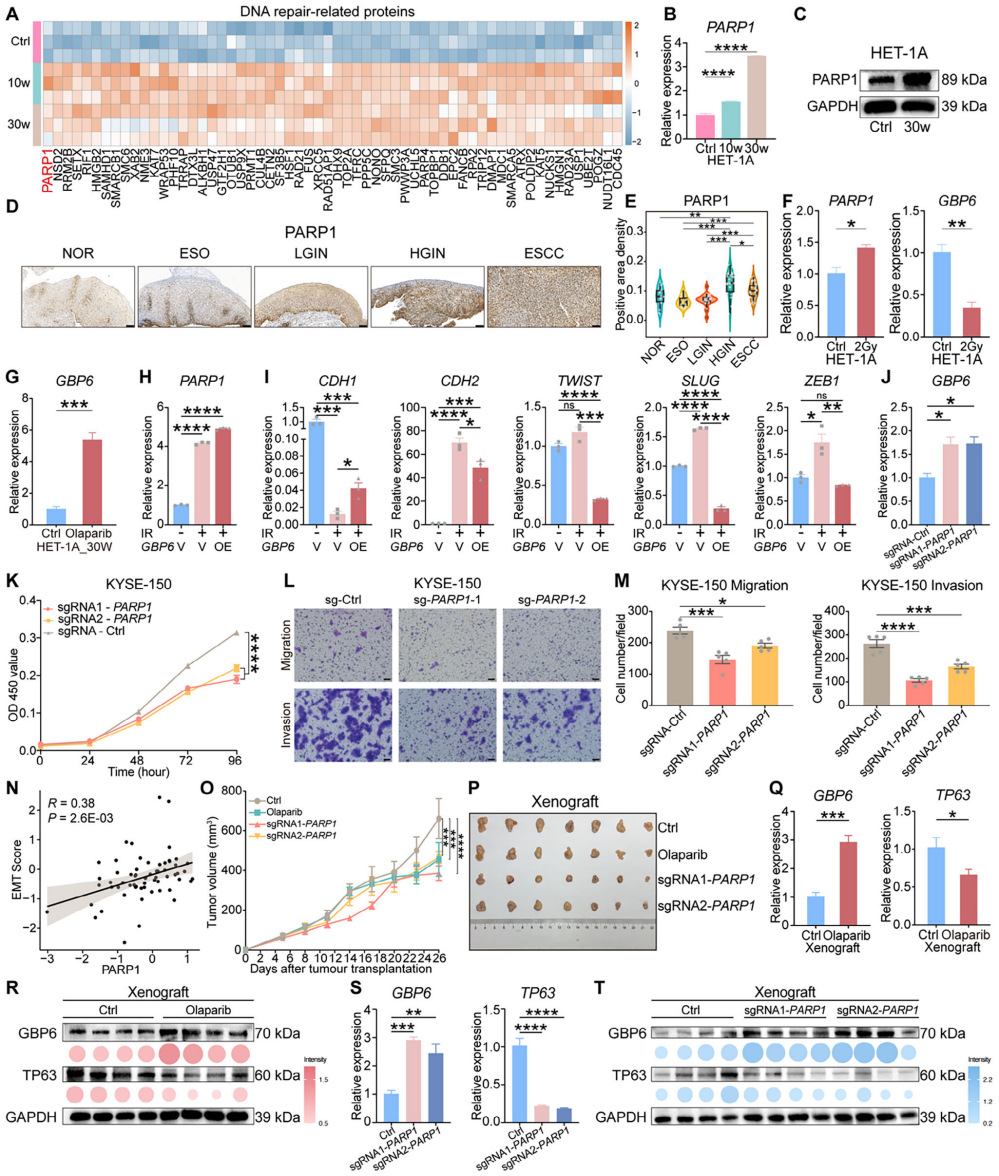
PARP1 inhibition rescues the gradual loss of GBP6 driven by DNA damage. (A) Heatmap shows the expression of DNA repair‐related proteins in the HET‐1A model. (B,C) Comparison of the mRNA (B) and protein (C) expression of PARP1 in the HET‐1A model. (D,E) Representative images (D) and positive area density (E) of immunohistochemistry staining for PARP1. Scale bar, 100 µm. (F) The mRNA expression levels of *PARP1* and *GBP6* were compared in HET‐1A cells between the control group and the irradiation exposure group. (G) Effect of the PARP1 inhibitor olaparib on *GBP6* expression in HET‐1A_30w cells. (H) The expression of *PARP1* significantly increased after irradiation‐induced DNA damage in HET‐1A_30w cells. IR: irradiation, V: vector, OE: overexpression. (I) *GBP6* overexpression partially reverses the DNA damage‐induced expression of EMT markers in HET‐1A_30w cells. (J) Effect of *PARP1* knockout on *GBP6* expression in KYSE‐150 cells. (K) Effects of *PARP1* knockout on the proliferation of KYSE‐150 cells. (L,M) Representative images (L) and statistics (M) show the effects of *PARP1* knockout on KYSE‐150 cell migration and invasion. Scale bar, 50 µm. (N) Pearson correlation analysis between PARP1 and EMT scores in L tissues. (O,P) Tumor volume (O) and general images (P) show the effects of *PARP1* knockout and PARP1 inhibitor olaparib in KYSE‐150 cells on their xenograft growth in mice. (Q) Effects of the PARP1 inhibitor olaparib on the expression of *GBP6* and *TP63* in mouse xenografts. (R) Western blotting assays demonstrate the effects of PARP1 inhibitor olaparib on GBP6 and TP63 protein expression in mouse xenografts. (S) Effects of *PARP1* knockout on the expression of *GBP6* and *TP63* in mouse xenografts. (T) Western blotting assays demonstrate the effects of *PARP1* knockout on GBP6 and TP63 protein expression in mouse xenografts. The *p* values were calculated by Student's t‐test (E–I,Q) or Dunnett's test (B,J,K,M,O,S). ^*^ *p* <0.05, ^**^ *p* <0.01, ^***^ *p* <0.001, ^****^ *p* <0.0001.

We next sought to investigate the causes of GBP6 loss during ESCC progression. First, we observed significant upregulation of proteins related to DNA damage repair in the HET‐1A model following chemical induction by NMBA (Figure 7A; Figure S8E). Notably, PARP1, a protein closely associated with DNA repair, was found to be significantly upregulated in both tissues and the HET‐1A model (Figures 4E and 7B,C). It was also confirmed in an independent NT‐Cohort of clinical tissue samples that PARP1 expression is gradually upregulated during ESCC progression (Figure 7D,E). We also found that PARP1 was significantly upregulated in tumor tissues, and was potentially associated with the prognosis of EC patients (Figure S10A–C). Therefore, we hypothesize that the loss of GBP6 is caused by DNA damage and is closely associated with PARP1. To test this hypothesis, we exposed normal esophageal epithelial cells HET‐1A to irradiation and found that *PARP1* was elevated, while *GBP6* was significantly reduced (Figure 7F). Additionally, treatment of HET‐1A_30w cells with the PARP1 inhibitor olaparib significantly increased *GBP6* expression (Figure 7G). Meanwhile, we found that *GBP6* overexpression partially reversed the DNA damage‐induced expression of EMT markers in HET‐1A_30w cells (Figure 7H,I). Taken together, these results indicate that the progressive loss of GBP6 is driven by DNA damage and that GBP6 loss serves as a major downstream oncogenic effector.

To further confirm the effect of PARP1 on malignant cell phenotype, we thus performed gene operation in ESCC cells and HET‐1A_30w cells (Figure S10D). We first found that *PARP1* knockout upregulated *GBP6* expression in ESCC cells (Figure 7J). The results also showed that *PARP1* knockout significantly suppressed cell proliferation, migration, and invasion (Figure 7K–M; Figure S10E–G). Additionally, we found that PARP1 expression was significantly and positively correlated with EMT scores (Figure 7N). We also explored the expression levels of genes involved in EMT in *PARP1* knockout ESCC and HET‐1A_30w cells, and found that *PARP1* knockout significantly decreased the mesenchymal genes (Figure S11A–C). Immunofluorescence staining also confirmed that *PARP1* knockout significantly suppressed the EMT (Figure S11D–I). We subsequently conducted in vivo functional experiments. The results demonstrated that PARP1 inhibition and knockout not only significantly inhibited the growth rate of ESCC cells transplanted in mice, but more importantly, rescued the progressive loss of GBP6 at both the mRNA and protein levels (Figure 7O–T). Taken together, these in vitro and in vivo experiments confirm that PARP1 inhibition effectively rescues the progressive loss of GBP6 caused by DNA damage, thereby suppressing ESCC progression.

To further investigate the molecular mechanism linking PARP1 and GBP6, we first identified potential transcription factors for GBP6 in Cistrome Data Browser [29] and selected the top candidates based on the Regulatory Potential Score (RPS). Among these TFs, we identified TP63, which is crucial for maintaining epithelial development and morphogenesis [30] and is closely associated with controlling the epigenetic and transcriptional patterns in ESCC cell lines (Figure S12A) [31]. We further analyzed the Chip‐seq data in Cistrome Data Browser [29] and found that TP63 can bind to the promoter region of *GBP6* (Figure S12B). We also observed a significant negative correlation between *TP63* and *GBP6* (Figure S12C). These findings suggest that TP63 acts as a potential transcriptional repressor of GBP6. Furthermore, it has been reported that TP63 participates in the downstream signaling of the DNA damage‐associated PARP1 pathway in carcinoma cells [32]. Consistently, we observed that *TP63* was significantly upregulated in both the malignantly transformed HET‐1A_30w cells and the irradiation‐induced DNA damage cell model, further confirming its involvement in DNA damage (Figure S12D,E). Our in vitro and in vivo experiments consistently showed that TP63 expression was significantly downregulated at both the mRNA and protein levels following the inhibition or knockout of PARP1 (Figure 7Q–T; Figure S12F). Collectively, we propose a novel mechanistic axis where PARP1 inhibition rescues the loss of GBP6 by suppressing its transcriptional repressor TP63.

## 3 Discussion

Integrated molecular analyses of genomic and transcriptomic data have illuminated the ESCC landscape, revealing key gene mutations, diagnostic and prognostic markers, and molecular subtypes [33, 34, 35, 36]. Proteins are more closely linked to biological phenotypes, making proteomic analysis a more intuitive and comprehensive strategy for characterizing the molecular profile of ESCC. Several studies have analyzed proteomes in tumor and normal tissues of ESCC, but protein profiling of precancerous lesions remains underexplored [16, 25]. The non‐amplifiable nature of proteins and the need for a certain amount of starting material for MS‐based proteomics make proteomic analysis of biopsy samples challenging under the premise of meeting clinical needs. Here, we constructed a refined and unbiased proteomic profile, comprising 4461 proteins covering the entire ESCC progression from ESO, MID, MOD, and SED to ESCC. Current studies predominantly isolate and analyze precancerous lesions from surgical specimens of ESCC patients. However, these samples may have already been influenced by adjacent tumor tissues and accumulated oncogenic alterations [37, 38, 39]. A distinct strength of this study lies in the collection of esophageal epithelial biopsy specimens, which authentically represent precancerous lesions for proteomic analysis. On the premise of meeting clinical needs, low‐input proteomics achieved proteomic characterization using extremely limited biopsy samples, with an average of over 1500 protein groups identified in each sample. In addition to epithelial lesion tissue, we also innovatively performed proteomic analysis on microscale adjacent non‐lesion tissue, which is of great significance for investigating the role of the local microenvironment in the progression of precancerous lesions. Taken together, this atlas addresses the critical gap in the lack of proteomic research on precancerous lesion progression and provides a valuable protein‐level resource for ESCC research.

We conducted DNB analysis to identify the tipping stage in ESCC progression [40, 41, 42]. After passing the tipping point, the system will enter another irreversible and stable state [20]. The results indicated that the MID serves as the tipping stage in L tissues, leading us to divide the progression into the preESCC phase and the eESCC phase. One strength of this study is that in identifying the critical turning stage, we not only conducted DNB analysis for L tissues but also innovatively incorporated the DNB results from N tissues into our comprehensive consideration. The identification of the critical turning stage is crucial for recognizing the clinical intervention window. A distinctive feature of this preliminarily stratification system is recognizing MOD as the initiation of a relatively malignant and irreversible state in ESCC progression, grouping MOD with SED and ESCC in the eESCC phase. These findings provide proteomic data support for the expert consensus on that LGIN (MID and MOD) patients are undertreated in clinical practice [3], and strongly merit further validation through longitudinal studies in future research. Consistently, a previous prospective study found that 50% of MOD patients developed to ESCC during 13 years of follow‐up [43]. Liu et al. also reported that the presence of a subset of LGIN patients shared a similar transcriptional context with HGIN and ESCC [8]. Our findings are corroborated by these previous epidemiological and transcriptomic data. An interaction network based on receptor‐ligand pairs was constructed at the MOD stage, revealing that the EMT may play a key role in ESCC progression. Collectively, these findings suggest that MOD patients may require early clinical intervention, and that further studies are needed to guide clinical treatment.

We also identified and validated early warning biomarkers for ESCC, with an SVM model constructed from a 7‐protein panel showing good performance in discriminating eESCC from preESCC. Notably, GBP6, an esophagus‐specific protein, emerged as an early key signature, highlighting its potential as an early biomarker and therapeutic target. Based on functional experiments, we have confirmed that GBP6 is a novel and promising early ESCC target. Mechanistic studies have revealed that DNA damage‐induced progressive loss of GBP6 promotes ESCC progression through cell cycle regulation and EMT. Nevertheless, no therapeutic drugs targeting GBP6 have been developed to date. It is well‐known that developing new anti‐tumor drugs is an extremely long, complex, and costly process, involving extensive experiments and lengthy clinical trial phases [44, 45]. Crucially, we demonstrated that PARP1 inhibition can rescue the progressive loss of GBP6 and prevent ESCC progression. Although Olaparib is commonly used as a PARP1 inhibitor, its effects on other PARPs may also contribute to the observed phenotypes [46]. Our genetic knockout experiments provide specific evidence to address this concern. We believe that through the combination of pharmacological data and genetic manipulation, the role of PARP1 can be confirmed. PARP1 is known to be involved in cell division, DNA repair, chromatin remodeling, and transcriptional regulation [24]. Several PARP inhibitors, such as olaparib, iniparib, and thioparib, have been developed for anti‐tumor therapies [47]. Therefore, this strategy is expected to substantially reduce the time and cost of drug development, thereby accelerating clinical translation. In addition, based on bioinformatic analyses, public data, and experimental evidence from in vitro and in vivo assays, we propose a novel PARP1‐TP63‐GBP6 mechanistic axis in ESCC progression. This axis requires and is highly worthy of further experimental validation and translation in future studies.

Despite key emerging findings, this study also has some limitations. First, since the proteomic atlas of ESCC progression was constructed using biopsy tissue samples from distinct individuals at different stages of progression, this dataset cannot fully capture the true temporal dynamics of protein molecular changes during ESCC progression, primarily due to confounding by inter‐individual biological variability. Consequently, the early warning model and critical turning point identification derived from the proteomic atlas are suggestive but require confirmation with longitudinal data. These early biomarkers also need to be validated by large‐scale longitudinal clinical samples or mouse models. Second, due to the inherent limitations of the cross‐sectional design, this study failed to distinguish between protein molecular progression trajectories in different ESCC subtypes. Third, due to the challenges commonly faced in this field of scarce specimens and limited materials, this study was unable to obtain sufficient longitudinal biopsy tissues to integrate genomic data. This has also resulted in the genomic landscape of the turning stage remaining unclear. It may require over a decade to conduct longitudinal follow‐up and collect tissue samples from patients progressing through esophagitis, mild dysplasia, moderate dysplasia, severe dysplasia, and ultimately to ESCC [43, 48]. This may require long‐term support from biobanks, close cooperation and good compliance from subjects, and complex ethical approval, as well as decades of time and resources investment. Future research should focus on the integration of multi‐omics data during the precancerous lesion stage by establishing long‐term longitudinal cohorts, collecting sufficient clinical biospecimens, and developing low‐input multi‐omics technologies.

In summary, our study is the first to systematically delineate the dynamic protein profiles associated with various stages of ESCC progression, revealing a significant loss of esophageal epithelial function and activation of oncogenic pathways in the early stage of ESCC progression. We preliminarily established both a novel stratification system for ESCC progression and a promising early protein warning model, which warrant further confirmation through integrated longitudinal multi‐omics studies in future research. We found that DNA damage‐induced progressive loss of GBP6 promotes ESCC progression by regulating the cell cycle and activating the EMT process. Encouragingly, we also identified PARP1 as an early ESCC target, and its inhibition can rescue the progressive loss of GBP6. Collectively, our findings provide new insights into the early prevention and intervention of ESCC.

## 4 Experimental Section

### 4.1 Sample Collection and Slides Preparation

We recruited 54 subjects with ESO, MID, MOD, SED, and ESCC at Yanting Cancer Hospital in Yanting, Sichuan Province, China, and collected a total of 71 biopsy samples. All endoscopic examinations were performed by well‐trained doctors in accordance with the Chinese guideline for the screening, early detection, and early treatment of esophageal cancer. The subjects underwent standard upper gastrointestinal endoscopy with iodine staining, and tissue biopsies were performed on focal suspected lesions. Two well‐trained pathologists independently read the biopsy slides for diagnosis, and any inconsistent results were resolved through discussion. Under the premise of meeting clinical diagnostic requirements, a limited portion of the biopsy tissue was collected for proteomic study. The sample size was determined primarily based on statistical power, the scarcity and collection challenges of samples, the properties of the biomarker discovery study [49], and with reference to similar studies [8, 9, 10, 11, 50, 51, 52]. Biopsy samples were processed into FFPE tissue blocks and cut into 5 µm thick sections. For LCM‐based proteomics, FFPE tissues mounted on MMI (Molecular Machines & Industries, Germany) membrane slides were dewaxed with xylene, washed with a gradient concentration of ethanol, and then subjected to hematoxylin and eosin (H&E) staining. In addition, we also collected an additional 80 independent biopsy samples for IHC to validate ESCC early tissue biomarkers at Nantong Tumor Hospital in Nantong, Jiangsu Province, China. All procedures complied with the guidelines of the Helsinki Declaration, and this study was approved by the Public Health and Nursing Research Ethics Committee of Shanghai Jiao Tong University School of Medicine and the Ethics Committee of Affiliated Tumor Hospital of Nantong University. Further details are presented in the Supporting Information.

### 4.2 Laser Capture Microdissection

The MMI membrane slides mounted FFPE tissues were loaded onto the stage of the MMI CellCut Laser Capture Microdissection System (Molecular Machines & Industries, Germany). Under the guidance of well‐trained pathologists, we then used a drawing tool to select the lesional regions and non‐lesional regions for microdissection, respectively. Details are presented in the Supporting Information.

### 4.3 Proteomic Analysis

For tissue samples, the preparation methods are mainly based on our previous work [53]. Briefly, to solubilize the proteins of the target tissues on the lids, we added lysis buffer directly to the lids of the tubes. The tissues were then incubated in a water bath and lysed at 60 °C for 2 h. To reduce and alkylate the proteins, tris(2‐chloroethyl)phosphate (TCEP, Sigma–Aldrich) and chloroacetamide (CAA, Sigma–Aldrich) were added and then subjected to incubate for 5 min at 95 °C. After cooling to room temperature, the magnetic beads (Thermo Fisher Scientific) were added to the system and thoroughly mixed with the proteins, and washed with ethanol after incubation. Trypsin (Thermo Fisher Scientific) was added to the system, and then subjected to incubate on the mixer at 1200 rpm at 37 °C for 4 h. The unlabeled, unfractionated peptides from tissue samples were analyzed on a trapped ion mobility spectrometer (TIMS) coupled to a time‐of‐flight mass spectrometer (timsTOF Pro, Bruker Daltonics) coupled to a high‐performance applied chromatographic system nanoElute (Bruker Daltonics) via a CaptiveSpray nano‐electrospray ion source (Bruker Daltonics). Peptides were loaded on an in‐house packed column (250 mm × 75 µm; 1.9 µm ReproSil‐Pur C18 beads, Dr. Maisch GmbH) using a 60‐min gradient (mobile phase A: 0.1% FA in water, mobile phase B: 0.1% FA in acetonitrile) of 2% to 80% mobile phase B at a flow rate of 300 nL/min. Raw files were processed using Spectronaut (Biognosys) with a project‐specific hybrid spectral library. For the proteomic analysis of malignantly transformed HET‐1A cell models, the unlabeled samples were analyzed on a trapped ion mobility spectrometer (TIMS) coupled to a time‐of‐flight mass spectrometer (timsTOF Pro 2, Bruker Daltonics) coupled to a high‐performance applied chromatographic system nanoElute 2 (Bruker Daltonics). Peptides were separated by a 60‐min gradient using an IonOpticks Aurora Ultimate CSI UHPLC column (25 cm × 75 µm, 1.7 µm C18, IonOpticks), and the column temperature was maintained at 50 °C. For the comparative proteomic analysis between control and *GBP6*‐overexpressing groups in HET‐1A_30w cells, the peptides were analyzed on a trapped ion mobility spectrometer (TIMS) coupled to a time‐of‐flight mass spectrometer (timsTOF Pro 2, Bruker Daltonics) coupled to an Evosep One liquid chromatography system (EvoSep Biosystems). Peptides were analyzed by 30 samples per day method (30 SPD) using an in‐house packed column (150 mm × 150 µm; 1.7 µm C18 beads, Dr. Maisch GmbH) at 50 °C. More details of proteomic analyses of tissue and cell samples are presented in the Supporting Information.

### 4.4 Data Analysis

Protein expression matrices were imported into R (version 4.1.1) for subsequent pre‐processing. The proteomic data were normalized using the median values, followed by a log2 transformation. The esophagus‐specific proteins, cancer‐related proteins, US Food and Drug Administration (FDA)‐approved drug targets, and potential drug targets were annotated by the Human Protein Atlas (HPA, https://www.proteinatlas.org/) database. Student's t test or Wilcoxon rank‐sum test was used to determine the differentially expressed proteins, depending on whether the expression data fit the normal distribution. Proteins with a significant level as *p* <0.05 and fold change (FC) >1.20 or FC <0.83 were considered as significantly upregulated or downregulated proteins, respectively. Functional enrichment analysis was performed using the online tool Metascape [54]. Gene set enrichment analysis (GSEA) was performed using clusterProfiler (v4.14.6) [55] R package with the Hallmark gene sets from MSigDB database [56]. The protein‐protein interaction (PPI) networks were constructed based on the STRING database (version 12.0) and visualized by Cytoscape (version 3.9.0). Functional subnetworks were extracted using Molecular Complex Detection (MCODE, version 2.0.3) in Cytoscape with default parameters. The MFuzz soft clustering was performed using “MFuzz” package (version 2.54.0) [57] in R. Dynamic network biomarker (DNB) analysis was applied to identify the tipping stage in ESCC progression [20]. The EMT score was the average mesenchymal protein expression minus the average epithelial protein expression. Transcription factor analysis was performed using the Cistrome Data Browser [29]. All the machine learning models in this study were constructed by “mlr3” package [58] (version 0.14.1) in R. We performed benchmarking to select the optimal machine learning model, and applied a recursive feature elimination algorithm to select the most informative features. Details are presented in the Supporting Information.

### 4.5 Immunohistochemistry

Briefly, the tissue slides were dewaxed and subjected to antigen retrieval. After blocking endogenous peroxidase with 3% H_2_O_2_ solution, non‐specific reactions were blocked with 3% bovine serum albumin (BSA) for 30 min at room temperature, followed by incubation with primary antibodies at 4 °C overnight. The antibodies used were as follows: PARP1 (Proteintech, 13371‐1‐AP, RRID: AB_2160459), ARPC5 (Abcam, ab51243, RRID: AB_2059963), C19orf53 (Santa Cruz Biotechnology, sc‐515133, RRID: AB_3712014), CCDC86 (Proteintech, 14947‐1‐AP, RRID: AB_2072318), GBP6 (Bioss, bs‐13304R, RRID: AB_3712015), GMPPB (Proteintech, 15094‐1‐AP, RRID: AB_2111064), PDCD6IP (Bioss, bs‐6767R, RRID: AB_3712016), and SF3A3 (Proteintech, 12070‐1‐AP, RRID: AB_2186372). The slides were then incubated with HRP‐conjugated secondary antibodies for 50 min at room temperature. After washing with PBS, the target proteins were visualized by diaminobenzidine (DAB) staining and counterstained with hematoxylin. IHC images were analyzed using AIpathwell software (Servicebio, Wuhan, China) according to the manufacturer's instructions. We quantified the expression level of target proteins according to the positive area density, and the positive area density was evaluated using the following formula: Positive area density = integrated optical density (IOD) / tissue area.

### 4.6 Functional Assays

ESCC cell lines KYSE‐150 (RRID: CVCL_1348) and TE‐1 (RRID: CVCL_1759) were purchased from Procell Life Science&Technology Co., Ltd. (12 January 2024, Wuhan, China). HET‐1A (RRID: CVCL_3702) cells were obtained from American Type Culture Collection (ATCC, USA). The malignantly transformed HET‐1A cell lines were obtained by continuous N‐nitrosomethylbenzylamine (NMBA) treatment in HET‐1A cells [59]. All cell lines were tested and confirmed to be free of contamination and authenticated using short tandem repeats (STR) profiling. Detailed information on functional assays such as cell irradiation, RNA extraction, and quantitative real‐time PCR, western blotting assay, cell proliferation assay, cell migration and invasion, immunofluorescence, colony formation and anchorage‐independent growth assay, and mouse xenograft assay are presented in the Supporting Information.

### 4.7 Statistical Analysis

Statistical analyses and visualizations were performed using R (version 4.1.1) and GraphPad Prism 10 unless otherwise stated. The details of statistical analysis can be found in the corresponding figure legends or table footnotes. The Venn diagrams were created by an online tool jvenn (https://jvenn.toulouse.inra.fr/app/index.html), and the upset plots were created by “UpSetR” package (version 1.4.0). Two‐group comparisons were performed by the Student's t test or the Wilcoxon rank‐sum test. One‐way analysis of variance (ANOVA) or two‐way ANOVA followed by Dunnett's post hoc test was used for multiple group comparisons, depending on the experimental design. All statistical tests were two‐sided, and the significance level was *p* <0.05 unless otherwise stated. Correlation analysis was tested using Pearson correlation coefficients unless otherwise stated. For survival analyses, the Kaplan‐Meier curves were used to show overall survival (OS) and disease‐free survival (DFS) probabilities, and the log‐rank test was applied to assess the statistical significance.

## Author Contributions

X.L., J.Y., M.G., and J.L. contributed equally to this work. H.W., X.G.L., C.L., and J.L. contributed to the study conceptualization, resources, design, and supervision. H.W., X.G.L., and C.L. contributed to funding acquisition. X.M.L., J.Y., M.G., J.B.L., X.L.H., H.Y., and C.L. contributed to the study methodology and formal analysis. X.M.L., J.Y., M.G., J.B.L., Q.Q.W., Y.Q.Z., M.T.C., and C.L. contributed to sample acquisition. X.M.L. and J.Y. contributed to the visualization and writing the original manuscript. H.W., X.G.L., and C.L. revised the manuscript. All authors reviewed and approved the final manuscript.

## Funding

This work was supported by the National Natural Science Foundation of China (NSFC) (82030099, 82373446, 81972820, 82574086), the National Key R&D Program of China (2022YFD2101500), the Shanghai Municipal Science and Technology Commission “Science and Technology Innovation Action Plan” Technical Standard Project (21DZ2201700), the Shanghai Municipal Science and Technology Commission “Science and Technology Innovation Action Plan” Natural Science Foundation Project (23ZR1435800, 23ZR1435900), Shanghai Public Health System Construction Three‐Year Action Plan (GWVI‐11.1‐43), the Science and Technology Commission of Shanghai Municipality (22DZ2303000), Shanghai Jiao Tong University Key Program of Medical Engineering (YG2021ZD01), and the Innovative Research Team of High‐level Local Universities in Shanghai.

## Conflicts of Interest

The authors declare no conflicts of interest.

## Supporting information




**Supporting File 1**: advs73729‐sup‐0001‐SuppMat.docx.

**Supporting File 2**: advs73729‐sup‐0002‐Supplementary Table_Production Data.xlsx.

## Data Availability

The data that support the findings of this study are available from the corresponding author upon reasonable request.
