# Supplementary material for: Spatio‐Temporal Proteomic Landscape Reveals Early Warning Signals of Esophageal Squamous Cell Carcinoma Progression
Source: Adv Sci (Weinh). 2026 Jan 8;13(16):e14343. doi: 10.1002/advs.202514343 (PMC13042397; doi:10.1002/advs.202514343)
Supplement: Supplementary file 1 — Supporting File 1: advs73729‐sup‐0001‐SuppMat.docx. [file ADVS-13-e14343-s002.docx]

Supporting Information

Spatio-Temporal Proteomic Landscape Reveals Early Warning Signals of Esophageal Squamous Cell Carcinoma Progression

Xumiao Li, Jie Yuan, Min Gao, Jibin Liu, Qinqin Wang, Yaqi Zhang, Mingtao Cao,

Xiaolin Hu, Hui Yang, Jun Li*, Chen Li*, Xiaoguang Li*, Hui Wang*

**Supplementary** **Materials and Methods**

**1. Cohorts and** **sample collection**

In this study, we recruited 54 subjects (Yanting cohort, YT-Cohort) with esophagitis (ESO), mild dysplasia (MID), moderate dysplasia (MOD), severe dysplasia (SED), or ESCC at Yanting Cancer Hospital in Yanting, Sichuan Province, a high incidence area for ESCC in China. Specifically, a total of 71 biopsy samples were collected, with 38 patients having biopsy samples collected at only one time point (time point 1), 15 patients at both two time points (time points 1, and 2), and one patient at all three time points (time points 1, 2, and 3). Finally, time point 1 consisted of 13 ESO, 21 MID, 6 MOD, 7 SED, and 7 ESCC biopsy samples, time point 2 consisted of 1 MID, 5 MOD, 7 SED, and 3 ESCC biopsy samples, and time point 3 consisted of 1 MOD biopsy sample. We also collected the demographics and clinical characteristics of the patients, including age, gender, body mass index (BMI), blood pressure, smoking, and alcohol consumption (**Table** S1, Supporting Information). In addition, we also collected an additional 80 independent biopsy samples (Nantong cohort, NT-Cohort) for immunohistochemistry (IHC) to validate ESCC early tissue biomarkers at Nantong Tumor Hospital in Nantong, Jiangsu Province, China, including 10 normal (NOR) esophageal squamous epithelium, 11 ESO, 17 low-grade intraepithelial neoplasia (LGIN), 22 high-grade intraepithelial neoplasia (HGIN) and 20 ESCC tissues (**Table** S2, Supporting Information). This study was approved by the Public Health and Nursing Research Ethics Committee of Shanghai Jiao Tong University School of Medicine and Affiliated Tumor Hospital of Nantong University.

**2. Slides preparation**

The biopsy samples were processed into FFPE tissue blocks and cut into 5 μm thick sections, which were then subjected to hematoxylin and eosin (H&E) staining for histopathologic diagnosis. Biopsy samples for proteomic analysis were mounted on MMI (Molecular Machines & Industries, Germany) membrane slides on the premise of meeting the biopsy samples required for histopathologic diagnosis. For FFPE tissues mounted on MMI membrane slides were dewaxed with xylene, washed with a gradient concentration of ethanol, and then subjected to H&E staining.

**3. Laser capture microdissection**

The MMI membrane slides mounted FFPE tissues were loaded onto the stage of the MMI CellCut Laser Capture Microdissection System (Molecular Machines & Industries, Germany). Under the guidance of well-trained pathologists, we then used a drawing tool to select the lesional regions and non-lesional regions for microdissection, respectively. Epithelial lesion tissue (ELT, L tissue) and adjacent non-lesion tissue (ANT, N tissue) with an area of approximately 0.1 mm^2^ were obtained from biopsy samples by LCM. Eventually, a total of 10 ESO_L, 19 MID_L, 11 MOD_L, 14 SED_L, 10 ESCC_L, 8 ESO_N, 17 MID_N, 8 MOD_N, 10 SED_N, and 5 ESCC_N trace tissues were collected on the MMI adhesive lids of tube (Molecular Machines & Industries, Germany). The target tissues were stored in the tubes with adhesive lids for later proteomic analysis.

**4. Proteomic data generation**

**4.1. Sample preparation**

For tissue samples, the preparation methods are mainly based on our previous work.^[1]^ Briefly, to solubilize the proteins of the target tissues on the lids, we added lysis buffer directly to the lids of tubes, where the lysis buffer was a mixture of 50 mM 4-(2-hydroxyethyl)piperazine-1-ethanesulfonic acid (HEPES, Sigma-Aldrich), 1% Sodium dodecyl sulfate (SDS, Thermo Fisher Scientific), 1% glycerol (Sigma-Aldrich), 1% Triton X-100 (Sigma-Aldrich), 1% Tween 20 (Sigma-Aldrich), 1% NP-40 (Sigma-Aldrich), 1% deoxycholate (Sigma-Aldrich), 5 mM ethylene diamine tetraacetic acid (EDTA, Sigma-Aldrich), and 50 mM NaCl (Sigma-Aldrich). The tissues were then incubated in a water bath and lysed at 60°C for 2 h. After centrifugation at 3,000 × g, to reduce and alkylate the proteins, tris(2-chloroethyl)phosphate (TCEP, Sigma-Aldrich) and chloroacetamide (CAA, Sigma-Aldrich) were added to the system at final concentrations of 10 mM and 40 mM, respectively, and then subjected to incubate for 5 min at 95°C. After cooling to room temperature, the magnetic beads (Thermo Fisher Scientific) were added to the system and thoroughly mixed with the proteins, and the protein-beads mixture was incubated on a mixer first at 1,500 rpm for 12 min and then at 1,200 rpm for 8 min at room temperature. Subsequently, the tubes were placed on a magnetic rack and the supernatant was removed and discarded. Next, the protein-bound beads were washed twice with 80% ethanol (Sinopharm Chemical Reagent) and then once with 100% ethanol. After the beads were air-dried, 250 ng trypsin (Thermo Fisher Scientific) dissolved in 100 mM ammonium bicarbonate (Sigma-Aldrich) was added to the system, and then subjected to incubate on the mixer at 1,200 rpm at 37°C for 4 h. After enzymatic digestion, the peptides were transferred to new tubes and acidified with 1% formic acid (FA, Thermo Fisher Scientific) for liquid chromatography-tandem mass spectrometry (LC-MS/MS).

For cell lines, cells were harvested by lysis buffer as described above, followed by heating at 95°C for 5 min and sonication in ice water (5 seconds on and 5 seconds off) for 2 min. The supernatant was then collected after centrifugation at 12,000 × g for 10 min and the protein concentration was detected using tryptophan-based fluorescence quantification method. Proteins were enzymatically digested as described above. Trypsin (Thermo Fisher Scientific) dissolved in 100 mM ammonium bicarbonate (Sigma-Aldrich) was added to the samples at 1:50 enzyme-to-protein ratio and digested at 37°C, 1,200 rpm for 4 h. After desalting using a C18 homemade StageTip, clean peptides were lyophilized and resuspended in 0.1% FA. Finally, the peptide concentration was determined using a Micro BCA Protein Assay Kit (Thermo Fisher Scientific) and 200 ng of peptides were subsequently analyzed by MS.

**4.2. LC-MS/MS analysis**

For the proteomic analysis of tissue samples, the unlabeled, unfractionated peptides from samples were analyzed on a trapped ion mobility spectrometer (TIMS) coupled to a time-of-flight mass spectrometer (timsTOF Pro, Bruker Daltonics) coupled to a high-performance applied chromatographic system nanoElute^®^ (Bruker Daltonics) via a CaptiveSpray nano-electrospray ion source (Bruker Daltonics). Peptides were loaded on an in-house packed column (250 mm × 75 μm; 1.9 μm ReproSil-Pur C18 beads, Dr. Maisch GmbH) using a 60-min gradient (mobile phase A: 0.1% FA in water, mobile phase B: 0.1% FA in acetonitrile) of 2% to 80% mobile phase B at a flow rate of 300 nL/min. The column temperature was heated and maintained at 60°C. The timsTOF Pro was operated in diaPASEF (parallel accumulation-serial fragmentation combined with data-independent acquisition) acquisition mode,^[2]^ and the parameters were as follows: *m/z* range from 100 to 1,700, ion mobility range from 0.75 to 1.35 1/K_0_, fragment analysis was subdivided into 64 × 26 Th precursor isolation windows from *m/z* 400 to 1,200 with 1 Th isolation width overlap, one mass-width window contained two mobility windows and 16 consecutive MS2 scans were distributed to one MS1 scan, and the collision energy was ramped linearly as a function of the mobility from 59 eV at 1/K_0_ = 1.6 Vs/cm^2^ to 20 eV at 1/K_0_ = 0.6 Vs/cm^2^.

For the proteomic analysis of malignantly transformed HET-1A cell models, the unlabeled samples were analyzed on a trapped ion mobility spectrometer (TIMS) coupled to a time-of-flight mass spectrometer (timsTOF Pro 2, Bruker Daltonics) coupled to a high-performance applied chromatographic system nanoElute^®^ 2 (Bruker Daltonics). Peptides were separated by a 60-min gradient using an IonOpticks Aurora Ultimate CSI UHPLC column (25 cm × 75 μm, 1.7 μm C18, IonOpticks) and the column temperature was maintained at 50°C. For the comparative proteomic analysis between control and *GBP6*-overexpressing groups in HET-1A_30w cells, the peptides were analyzed on a trapped ion mobility spectrometer (TIMS) coupled to a time-of-flight mass spectrometer (timsTOF Pro 2, Bruker Daltonics) coupled to an Evosep One liquid chromatography system (EvoSep Biosystems). Peptides were analyzed by 30 samples per day method (30 SPD) using an in-house packed column (150 mm × 150 μm; 1.7 μm C18 beads, Dr. Maisch GmbH) at 50°C. Other parameters were in line with the above.

**4.3. Database searching for MS data**

For tissue samples, MS raw files were processed using Spectronaut^®^ (version 17, Biognosys). A project-specific hybrid spectral library containing 51,383 precursors, 41,156 peptides, and 4,791 protein groups was generated by Pulsar algorithm using 32 DIA runs and 5 mix (tissue sample mixture) DIA runs. All of the DIA runs were analyzed by Spectronaut^®^ (version 17, Biognosys) with default parameters against this project-specific hybrid spectral library. In brief, enzyme specificity was set as trypsin/P, and allowing a maximum of two mis-cleavage sites. Carbamidomethyl of cysteine was defined as a fixed modification, and oxidation of methionine and acetylation of the protein N-term were defined as variable modifications. The peptide length of 7-52 amino acids was allowed for the search. The false discovery rate (FDR) was controlled at 1% at both the peptide and protein group level. For cell lines, raw files were analyzed in Spectronaut^®^ (version 19, Biognosys) with a directDIA method and searched against the UniProt human database (2023 release, 20,593 entries). Other parameters were in line with the above.

**5. Proteomic** **data analysis**

**5.1. Proteomic data pre-processing**

Protein expression matrices were obtained from Spectronaut as described above and then imported into R (version 4.1.1) for subsequent pre-processing. The proteomic data were normalized using the median values, followed by a log_2_ transformation. For subsequent analysis of tissue samples, proteins that were quantified in at least one quarter of the samples were retained. In the case of the proteomic data from malignantly transformed HET-1A cell lines, proteins quantified in at least two-thirds of the samples were used for further analysis. The Partial Least Squares Discriminant Analysis (PLSDA) was performed using the “plsda” function of “mixOmics” package (version 6.18.1) in R.

**5.2. Protein annotation and** **subcellular distribution**

The esophagus-specific proteins, cancer-related proteins, US Food and Drug Administration (FDA)-approved drug targets and potential drug targets were annotated by the Human Protein Atlas (HPA, https://www.proteinatlas.org/) database. The subcellular distribution of proteins was analyzed by the Database for Annotation, Visualization and Integrated Discovery (DAVID, https://david.ncifcrf.gov/) online tool with default parameters. The epithelial-mesenchymal transition (EMT) pathway and associated proteins were downloaded from the Hallmark gene set via MSigDb (https://www.gsea-msigdb.org/gsea/msigdb/).

**5.3. Differentially expressed proteins**

For the analysis of differentially expressed proteins (DEPs), Student’s t test or the Wilcoxon rank-sum test was employed to determine the DEPs between two comparison groups, depending on whether the expression data fit the normal distribution. Proteins with the significant level as *P* < 0.05 and fold change (FC) > 1.20 or FC < 0.83 were considered as significantly upregulated or downregulated proteins, respectively.

**5.4. Functional enrichment analysis**

Functional enrichment analysis was performed using an online enrichment analysis tool Metascape^[3]^ (http://metascape.org) under the default parameters (minimum overlap = 3, *P* value cutoff = 0.01, minimum enrichment = 1.5). Gene set enrichment analysis (GSEA) was performed using clusterProfiler (v4.14.6)^[4]^ R package and the Hallmark gene sets were downloaded from MSigDB database.^[5]^

**5.5. Protein-protein interaction (PPI) network construction**

The protein-protein interaction (PPI) networks were constructed based on the STRING database (version 12.0, https://string-db.org/) and visualized by Cytoscape (version 3.9.0, https://cytoscape.org/). The transcription factors (TFs) were annotated by the Transcriptional Regulatory Relationships Unraveled by Sentence-based Text mining (TRRUST, version 2, https://www.grnpedia.org/trrust/) database.^[6]^ The functional subnetworks were extracted from the PPI networks by Molecular COmplex Detection (MCODE, version 2.0.3) in Cytoscape (version 3.9.0) using the default parameters. The core transcriptional regulatory network was constructed based on TF-target protein pairs in TRRUST (version 2) database.^[6]^ In addition, to explore proteins that interact with informative features, we constructed a PPI network based on our data and the Human Reference Interactome (HuRI, http://interactome-atlas.org) database.^[7]^

**5.6. Survival analysis and** **mRNA expression analysis in TCGA cohort**

To assess whether mRNA expression levels are significantly associated with prognosis in esophageal cancer patients, the Gene Expression Profiling Interactive Analysis (GEPIA, http://gepia.cancer-pku.cn/) database^[8]^ was employed for survival analysis based on the TCGA cohort. The analysis of whether PARP1 protein expression is associated with prognosis in esophageal cancer patients was performed using The Cancer Proteome Atlas (TCPA, https://www.tcpaportal.org/) database.^[9]^ In addition, PARP1 and GBP6 mRNA expression in normal and tumor tissues was assessed by GEPIA (http://gepia.cancer-pku.cn/) database^[8]^ and matching with TCGA and the Genotype-Tissue Expression (GTEx) data.

**5.7. Abundance patterns of proteins**

To comprehend the alterations in abundance patterns of shared proteins between L and N tissues at each stage, we conducted an analysis utilizing a published methodology as reference.^[10]^ Initially, within each stage, the shared proteins between L and N tissues were arranged based on their mean abundance values in L and N tissues, respectively. Subsequently, utilizing the abundance order from high to low, proteins were equally divided into six groups, defined as L1-L6 for L tissues and N1-N6 for N tissues. Lastly, chord diagram was employed to visualize the alterations in protein abundance between L and N tissues.

**5.8. Mfuzz soft clustering**

The Mfuzz soft clustering was performed using “Mfuzz” package (version 2.54.0)^[11]^ in R (version 4.1.1). Only the proteins with a membership value greater than 0.5 were deemed core proteins of this cluster and utilized for subsequent functional enrichment analysis.

**5.9. Dynamic network biomarker (DNB) analysis**

To identify the tipping stage in ESCC progression for early diagnosis and prevention, we applied the DNB method to analyze proteomic data. DNB analysis has been used in many studies to identify the critical state or tipping point of complex diseases.^[12-15]^ According to nonlinear dynamical theory, the biological system will fluctuate drastically before critical transition, and the presence of a group of molecules (i.e., DNB members) that fluctuate strongly and collectively in the expression data indicates the tipping point for system. After passing the tipping point, the biological system will enter another irreversible and stable state.^[16]^ DNB proteins must simultaneously fulfil the following three conditions: (1) The expression of DNB proteins fluctuated drastically, i.e., the average coefficient of variation increased significantly (CV_in_); (2) High correlation between DNB proteins, i.e., the average Pearson’s correlation coefficient (absolute value) increased markedly (PCC_in_); (3) The average Pearson’s correlation coefficient (absolute value) between DNB proteins and non-DNB proteins decreased significantly (PCC_out_).^[12-16]^

Taking these three conditions into account, the composite index (CI) was created and was able to approximate the quantification of this methodology with the following formula:^[14]^

$$CI=\mathrm{CV}_{\mathrm{in}} \times\frac{\mathrm{PCC}_{\mathrm{in}}}{\mathrm{PCC}_{\mathrm{out}}}$$

When the CI reaches its maximum value, it indicates that the system at that time point is at a tipping point or critical stage. Therefore, we first selected the proteins with the top 50% coefficient of variation for subsequent analyses. Next, clustering selected proteins resulted in protein modules and CI values were calculated for each module. Finally, the protein modules were compared to identify the module with the maximum CI value, the proteins within this module are the DNB proteins, and the corresponding time point is the tipping point or critical stage.

In addition, the PPI networks between DNB proteins and DEPs were constructed using the STRING database (version 12.0, https://string-db.org/) and visualized by Cytoscape (version 3.9.0, https://cytoscape.org/).

**5.10. iTALK analysis**

The interaction between L and N tissues of MOD stage was investigated by iTALK analysis using the “iTALK” package (version 0.1.0)^[17]^ in R (version 4.1.1), a method for characterizing and illustrating the ligand-receptor mediated intercellular cross-talk signals in multicellular systems. The ligand-receptor interaction pairs included cytokine, immune checkpoint, growth factor, and other ligand-receptor pairs based on the iTALK database. The top 25 ligand-receptor pairs were used to construct a ligand-receptor interaction network.

**5.11. Epithelial-mesenchymal transition (EMT) score**

The EMT score was calculated as previously described.^[18]^ Briefly, the EMT score was the value resulting from the average expression of mesenchymal (M) protein minus the average expression of epithelial (E) protein. The EMT signatures for tumors and cell lines were obtained from a previous study.^[19]^ In the present study, the EMT scores for tissue samples and malignantly transformed HET-1A cell lines were calculated using the EMT signatures for tumors and cell lines, respectively. All missing values were removed from the calculation.

**5.12. Machine learning model construction**

All the machine learning models in this study were constructed by “mlr3” package^[20]^ (version 0.14.1) in R (version 4.1.1). Missing values were imputed using “missForest” package (version 1.5) in R with default parameters. Based on the expression data of 234 DEPs in L tissues between 29 preESCC samples and 35 eESCC samples, we performed benchmarking with 10-fold cross-validation, repeated 10 times for several machines learning algorithms, including generalized linear models with elastic net regularization (glmnet), k-nearest-neighbor (kknn), linear discriminant analysis (lda), logistic regression (log_reg), naive bayes (naive_bayes), single layer neural network (nnet), random classification forest (ranger), decision trees (rpart), support vector machine (SVM), and extreme gradient boosting (xgboost). The performance of machine learning models was assessed by two metrics, the area under the curve (AUC) value of the receiver operating characteristic (ROC) curve and the Logloss value. A higher AUC value and a lower Logloss value indicate superior performance of machine learning model. Ultimately, the SVM algorithm was selected for further study due to its better performance as evaluated by AUC and Logloss value. Next, we used the feature importance based SVM-recursive feature elimination (SVM-RFE) algorithm to shrink the number of features and obtain the most informative features. When the number of features included in the model was recursively eliminated to 61 features, the SVM model achieved an AUC of 1.00. We further shrunk the number of features included in the model, and ultimately identified the seven most informative features (CCDC86, GBP6, PDCD6IP, C19orf53, SF3A3, GMPPB, and ARPC5) for constructing the final model. Finally, based on the most informative features, we constructed a 10-fold cross-validation and repeated 10 times SVM model to discriminate the patients with early ESCC. In addition, to validate the performance of the machine learning model in external independent cohorts, we extract the expression data of seven features from Li et al. cohort 1^[21]^ and the expression data of six features (expression data of C19orf53 is not available) from Li et al. cohort 2,^[22]^ respectively. Specifically, Li et al. cohort 1^[21]^ contained 94 ESCC tumor tissue samples and 24 non-tumor esophageal tissue samples. And Li et al. cohort 2^[22]^ contained 114 normal epithelial tissue samples and 466 ESCC samples.

**6. Functional assays**

**6.1. Cell lines and cell culture**

ESCC cell lines KYSE-150 (RRID: CVCL_1348) and TE-1 (RRID: CVCL_1759) were purchased from Procell Life Science&Technology Co., Ltd. (12 January 2024, Wuhan, China). HET-1A (RRID: CVCL_3702) cells were obtained from American Type Culture Collection (ATCC, USA). All cell lines were tested and confirmed to be free of contamination and authenticated using short tandem repeats (STR) profiling. The malignantly transformed HET-1A cell lines were obtained by continuous N-nitrosomethylbenzylamine (NMBA)-treatment in HET-1A cells for 10 or 30 weeks, as previously reported.^[23]^ KYSE-150 and TE-1 cells were cultured in RPMI 1640 medium (Gibco) with 10% FBS (Gibco) at 37°C under 5% CO_2_ conditions. The malignantly transformed HET-1A cell lines were cultured in Bronchial epithelial cell basal medium (BEGM) with all the additives (MeilunBio) at 37°C under 5% CO_2_ conditions.

**6.2. Cell irradiation**

The HET-1A cells were irradiated three times at 48-hour intervals using an RS-2000 Pro system (Rad Source) with 2 Gy of 160 kV, 25 mA X-rays for 104 s (0.3 mm filter).

**6.3. Establishment of cell lines with altered *GBP6* expression**

To establish TE-1 cells and malignantly transformed HET-1A cells with overexpression of *GBP6*, the *GBP6* sequences (CGCAAATGGGCGGTAGGCGTG) were first cloned into the pLX304-Blast-V5 vector (NovoPro, Shanghai, China). The vector was then co-transfected with a packing vector into 293T cells to generate lentivirus. The cell lines were then infected with the lentivirus for 48 h to achieve stable *GBP6* overexpression. Stable cells were screened with puromycin for 1-2 weeks. To generate *GBP6*-knockdown HET-1A cells, a short interfering RNA (siRNA) target human *GBP6* was purchased from Genomeditech (Shanghai, China). The HET-1A cells were cultured in six-well plates until they reached 70% confluence. The cells were then transfected with 50 nM siRNA-*GBP6* or a negative control using Lipofectamine 3000 (Thermo Fisher Scientific) according to the manufacturer’s instructions. The efficiency of *GBP6* overexpression and knockdown was assessed by Western blotting assays.

**6.4. Establishment of cell lines with altered *PARP1* expression**

To generate *PARP*1-knockout ESCC cells (KYSE-150 and TE-1) and malignantly transformed HET-1A cells, the CRISPR/Cas9 system was applied to introduce *PARP1* deletion. The sequences of *PARP1*-sgRNA were designed using the CRISPR designing tool and synthesized by BioSune Biotechnology (Shanghai, China). The negative control sequence was ACGGAGGCTAAGCGTCGCAA, and the knockdown target sequences were GTCCAACAGAAGTACGTGCA and GGTGCGCCTGTCCAAGAAGA. *PARP1*-sgRNA was cloned into the LentiCRISPR v2 vector (52961, Addgene, Watertown, MA) and co-transfected with the packaging vector into 293T cells to generate lentivirus. To obtain knockout clones, ESCC cells (KYSE-150 and TE-1) and malignantly transformed HET-1A cells were transfected with lentivirus for 48 h. Stable cells were screened with puromycin for 1-2 weeks. The efficiency of *PARP1* knockout was assessed by Western blotting assays.

**6.5. RNA extraction and** **quantitative real-time PCR**

Total RNA was extracted from ESCC cells (KYSE-150 and TE-1) and malignantly transformed HET-1A cells using the TRIzol Reagent and Direct-zol™ RNA Miniprep Plus Kit (Zymo Research) according to the manufacturer’s instructions. This was followed by reverse transcription using the PrimeScript™ RT reagent Kit (TaKaRa). Quantitative real-time PCR was performed using SYBR green (Selleckchem) with a CFX Opus 384 Real-Time PCR System (BIO-RAD). Relative mRNA expression levels were determined by the ΔΔC_t_ method and normalized to *GAPDH* mRNA level. The primers used in this study are shown in **Table** S15, Supporting Information.

**6.6. Western blotting assay**

Total proteins were extracted from cells using RIPA lysis buffer (EpiZyme), and the protein concentrations were measured using the Detergent Compatible Bradford Protein Assay Kit (Beyotime). The extracted proteins were then separated by SDS-PAGE (EpiZyme) and transferred onto PVDF membranes (Cytiva). After blocking with BSA, the membranes were incubated overnight at 4°C with primary antibodies against PARP1 (Cell Signaling Technology, 9532S, RRID: AB_659884), GBP6 (Bioss, bs-13304R, RRID: AB_3712015; Aladdin Scientific, Ab191420, RRID: AB_3717803) or TP63 (Proteintech, 86154-1-RR, RRID: AB_3717804), followed by incubation with secondary antibody (Cell Signaling Technology, 7074S, RRID: AB_2099233) for 2 h at room temperature. The protein signals were visualized using a SuperSignal™ West Pico PLUS Stable Peroxide Solution (Thermo Fisher Scientific) in Tanon-5200 Chemiluminescent Imaging System (Tanon Science & Technology). GAPDH was used as the housekeeping control.

**6.7. Cell proliferation assay**

Cells were seeded into 96-well plates (1,000 cells/well for ESCC and HET-1A_30w cells, 2,000 cells/well for HET-1A cells), and cell proliferation was assessed using the Cell Counting Kit-8 (MeilunBio) according to the manufacturer’s protocol. Proliferation was determined by measuring the absorbance at a wavelength of 450 nm for 5 consecutive time points using a BioTek Synergy H1 Microplate Reader (Agilent).

**6.8. Cell migration and invasion**

Cell migration and invasion of ESCC and malignantly transformed HET-1A cells were assessed using Transwell assays (8.0-μm-pore Transwell, Corning). For the migration assays, cells were seeded into the upper chambers containing serum-free medium, while the lower chambers were filled with medium containing 10% serum. After 12 h, migrated cells were fixed with paraformaldehyde (Servicebio) and stained with crystal violet (Beyotime). For invasion assays, the procedure was similar, expect the upper chambers were coated with Matrigel (R&D Systems). Random fields were selected for analysis using ImageJ software.

**6.9. Immunofluorescence**

Cells were seeded onto coverslips and fixed with 4% paraformaldehyde (Servicebio), followed by permeabilization with Triton X-100 (Beyotime). After blocking with BSA, the coverslips were incubated with primary antibodies against VIM (Cell Signaling Technology, 5741S, RRID: AB_10695459), E-cadherin (Cell Signaling Technology, 3195S, RRID: AB_2291471), and N-cadherin (Cell Signaling Technology, 13116S, RRID: AB_2687616) at 4°C overnight, followed by washing and incubation with secondary antibody (Thermo Fisher Scientific). Then, cells were stained with 4,6-diamidino-2-phenylindole (DAPI, Beyotime) for 3 min at room temperature. Images were captured using a laser scanning confocal microscopy (Leica) and analyzed using the ImageJ software.

**6.10. Colony formation and anchorage-independent growth assay**

For colony formation assay, TE-1 (1,000 cells/well) and malignantly transformed HET-1A cells (HET-1A_30w, 1,000 cells/well) were seeded in six-well plates and cultured for 10 days. After washed with PBS, the cells were fixed with paraformaldehyde (Servicebio) and stained with crystal violet (Beyotime). Images were captured by ChemiDoc Imaging System (BIO-RAD) and counted using the ImageJ software. For anchorage-independent growth assay, 1.2% and 0.7% agarose gels (Beyotime) were prepared and maintained at 42°C. The 1.2% agarose gels were mixed with 2× medium and added to the bottom layer of the six-well plates. After solidification, the 0.7% agarose gels were mixed with cell suspension (1,000 cells) and added to the upper layer. As mentioned above, the plates were then incubated for 25 days. Subsequently, the samples were stained with Nitrotetrazolium Blue chloride (NBT, MeilunBio). Images were captured by ChemiDoc Imaging System (BIO-RAD), and the colonies were counted using ImageJ software.

**6.11. Mouse xenograft assay**

BALB/c-nu mice (8 weeks old) were purchased from SLAC Laboratory Animal Co., Ltd. (Shanghai, China). After being washed and resuspended in 100 μL RPMI 1640 medium (Gibco), KYSE-150 (2 × 10^6^ cells) with or without *PARP1* knockout were subcutaneously injected into the left lower lumbar abdomen of the mice. Tumor volume was measured every 3 days with a caliper and calculated as follows: Tumor volume = length × width^2^ × 0.5. The mice were sacrificed 26 days after treatment. In addition, we also performed *in vivo* antitumor assays with olaparib, an inhibitor of PARP1. ESCC cells were subcutaneously injected into the left lower lumbar abdomen of nude mice as described above. Olaparib was dissolved in Cosolvent (2% DMSO, 40% PEG400, 5% Tween 80, and 53% Saline). When the tumor volume reached 100 mm^3^, the mice were treated with olaparib (50 mg/kg/d) by intraperitoneal injection. Four samples were randomly selected from each group to validate the expression levels of key molecules. The procedures for quantitative real-time PCR and Western blotting were consistent with those described above. All animal experiments were performed in accordance with the guidelines approved by the Institutional Animal Care and Use Committee of Shanghai Jiao Tong University School of Medicine.

**References**

[1] L. Gu, X. Li, W. Zhu, Y. Shen, Q. Wang, W. Liu, J. Zhang, H. Zhang, J. Li, Z. Li, Z. Liu, C. Li, H. Wang, Ultrasensitive proteomics depicted an in-depth landscape for the very early stage of mouse maternal-to-zygotic transition. *J. Pharm. Anal.* **2023**, *13*, 942.

[2] F. Meier, A.-D. Brunner, M. Frank, A. Ha, I. Bludau, E. Voytik, S. Kaspar-Schoenefeld, M. Lubeck, O. Raether, N. Bache, R. Aebersold, B. C. Collins, H. L. Röst, M. Mann, diaPASEF: parallel accumulation–serial fragmentation combined with data-independent acquisition. *Nat. Methods* **2020**, *17*, 1229.

[3] Y. Zhou, B. Zhou, L. Pache, M. Chang, A. H. Khodabakhshi, O. Tanaseichuk, C. Benner, S. K. Chanda, Metascape provides a biologist-oriented resource for the analysis of systems-level datasets. *Nat. Commun.* **2019**, *10*, 1523.

[4] G. Yu, L.-G. Wang, Y. Han, Q.-Y. He, clusterProfiler: an R Package for Comparing Biological Themes Among Gene Clusters. *OMICS* **2012**, *16*, 284.

[5] A. Subramanian, P. Tamayo, V. K. Mootha, S. Mukherjee, B. L. Ebert, M. A. Gillette, A. Paulovich, S. L. Pomeroy, T. R. Golub, E. S. Lander, J. P. Mesirov, Gene set enrichment analysis: A knowledge-based approach for interpreting genome-wide expression profiles. *Proc Natl Acad Sci U S A.* **2005**, *102*, 15545.

[6] H. Han, J.-W. Cho, S. Lee, A. Yun, H. Kim, D. Bae, S. Yang, C. Y. Kim, M. Lee, E. Kim, S. Lee, B. Kang, D. Jeong, Y. Kim, H.-N. Jeon, H. Jung, S. Nam, M. Chung, J.-H. Kim, I. Lee, TRRUST v2: an expanded reference database of human and mouse transcriptional regulatory interactions. *Nucleic Acids Res.* **2018**, *46*, D380.

[7] K. Luck, D.-K. Kim, L. Lambourne, K. Spirohn, B. E. Begg, W. Bian, R. Brignall, T. Cafarelli, F. J. Campos-Laborie, B. Charloteaux, D. Choi, A. G. Coté, M. Daley, S. Deimling, A. Desbuleux, A. Dricot, M. Gebbia, M. F. Hardy, N. Kishore, J. J. Knapp, I. A. Kovács, I. Lemmens, M. W. Mee, J. C. Mellor, C. Pollis, C. Pons, A. D. Richardson, S. Schlabach, B. Teeking, A. Yadav, M. Babor, D. Balcha, O. Basha, C. Bowman-Colin, S.-F. Chin, S. G. Choi, C. Colabella, G. Coppin, C. D’Amata, D. De Ridder, S. De Rouck, M. Duran-Frigola, H. Ennajdaoui, F. Goebels, L. Goehring, A. Gopal, G. Haddad, E. Hatchi, M. Helmy, Y. Jacob, Y. Kassa, S. Landini, R. Li, N. van Lieshout, A. MacWilliams, D. Markey, J. N. Paulson, S. Rangarajan, J. Rasla, A. Rayhan, T. Rolland, A. San-Miguel, Y. Shen, D. Sheykhkarimli, G. M. Sheynkman, E. Simonovsky, M. Taşan, A. Tejeda, V. Tropepe, J.-C. Twizere, Y. Wang, R. J. Weatheritt, J. Weile, Y. Xia, X. Yang, E. Yeger-Lotem, Q. Zhong, P. Aloy, G. D. Bader, J. De Las Rivas, S. Gaudet, T. Hao, J. Rak, J. Tavernier, D. E. Hill, M. Vidal, F. P. Roth, M. A. Calderwood, A reference map of the human binary protein interactome. *Nature* **2020**, *580*, 402.

[8] Z. Tang, C. Li, B. Kang, G. Gao, C. Li, Z. Zhang, GEPIA: a web server for cancer and normal gene expression profiling and interactive analyses. *Nucleic Acids Res.* **2017**, *45*, W98.

[9] M.-J. M. Chen, J. Li, Y. Wang, R. Akbani, Y. Lu, G. B. Mills, H. Liang, TCPA v3.0: An Integrative Platform to Explore the Pan-Cancer Analysis of Functional Proteomic Data *. *Mol. Cell. Proteomics* **2019**, *18*, S15.

[10] K. Yan, B. Bai, Y. Ren, B. Cheng, X. Zhang, H. Zhou, Y. Liang, L. Chen, J. Zi, Q. Yang, Q. Zhao, S. Liu, The Comparable Microenvironment Shared by Colorectal Adenoma and Carcinoma: An Evidence of Stromal Proteomics. *Front. Oncol.* **2022**, *12*, 848782.

[11] L. Kumar, E. F. M, Mfuzz: a software package for soft clustering of microarray data. *Bioinformation* **2007**, *2*, 5.

[12] Z. Fang, X. Han, Y. Chen, X. Tong, Y. Xue, S. Yao, S. Tang, Y. Pan, Y. Sun, X. Wang, Y. Jin, H. Chen, L. Hu, L. Hui, L. Li, L. Chen, H. Ji, Oxidative stress-triggered Wnt signaling perturbation characterizes the tipping point of lung adeno-to-squamous transdifferentiation. *Signal Transduct. Target. Ther.* **2023**, *8*, 16.

[13] S. Tang, Y. Xue, Z. Qin, Z. Fang, Y. Sun, C. Yuan, Y. Pan, Y. Zhao, X. Tong, J. Zhang, H. Huang, Y. Chen, L. Hu, D. Huang, R. Wang, W. Zou, Y. Li, R. K. Thomas, A. Ventura, K.-K. Wong, H. Chen, L. Chen, H. Ji, Counteracting lineage-specific transcription factor network finely tunes lung adeno-to-squamous transdifferentiation through remodeling tumor immune microenvironment. *Natl. Sci. Rev.* **2023**, *10*, nwad028.

[14] Z. Jiang, L. Lu, Y. Liu, S. Zhang, S. Li, G. Wang, P. Wang, L. Chen, SMAD7 and SERPINE1 as novel dynamic network biomarkers detect and regulate the tipping point of TGF-beta induced EMT. *Sci. Bull.* **2020**, *65*, 842.

[15] B. Yang, M. Li, W. Tang, W. Liu, S. Zhang, L. Chen, J. Xia, Dynamic network biomarker indicates pulmonary metastasis at the tipping point of hepatocellular carcinoma. *Nat. Commun.* **2018**, *9*, 678.

[16] L. Chen, R. Liu, Z.-P. Liu, M. Li, K. Aihara, Detecting early-warning signals for sudden deterioration of complex diseases by dynamical network biomarkers. *Sci Rep* **2012**, *2*, 342.

[17] W. Yuanxin, W. Ruiping, Z. Shaojun, S. Shumei, J. Changying, H. Guangchun, W. Michael, A. Jaffer, F. Andy, W. Linghua, iTALK: an R Package to Characterize and Illustrate Intercellular Communication. *bioRxiv* **2019**, 507871.

[18] R. D. Burk, Z. Chen, C. Saller, K. Tarvin, A. L. Carvalho, C. Scapulatempo-Neto, H. C. Silveira, J. H. Fregnani, C. J. Creighton, M. L. Anderson, P. Castro, S. S. Wang, C. Yau, C. Benz, A. G. Robertson, K. Mungall, L. Lim, R. Bowlby, S. Sadeghi, D. Brooks, P. Sipahimalani, R. Mar, A. Ally, A. Clarke, A. J. Mungall, A. Tam, D. Lee, E. Chuah, J. E. Schein, K. Tse, K. Kasaian, Y. Ma, M. A. Marra, M. Mayo, M. Balasundaram, N. Thiessen, N. Dhalla, R. Carlsen, R. A. Moore, R. A. Holt, S. J. M. Jones, T. Wong, A. Pantazi, M. Parfenov, R. Kucherlapati, A. Hadjipanayis, J. Seidman, M. Kucherlapati, X. Ren, A. W. Xu, L. Yang, P. J. Park, S. Lee, B. Rabeno, L. Huelsenbeck-Dill, M. Borowsky, M. Cadungog, M. Iacocca, N. Petrelli, P. Swanson, A. I. Ojesina, X. Le, G. Sandusky, S. N. Adebamowo, T. Akeredolu, C. Adebamowo, S. M. Reynolds, I. Shmulevich, C. Shelton, D. Crain, D. Mallery, E. Curley, J. Gardner, R. Penny, S. Morris, T. Shelton, J. Liu, L. Lolla, S. Chudamani, Y. Wu, M. Birrer, M. D. McLellan, M. H. Bailey, C. A. Miller, M. A. Wyczalkowski, R. S. Fulton, C. C. Fronick, C. Lu, E. R. Mardis, E. L. Appelbaum, H. K. Schmidt, L. A. Fulton, M. G. Cordes, T. Li, L. Ding, R. K. Wilson, J. S. Rader, B. Behmaram, D. Uyar, W. Bradley, J. Wrangle, A. Pastore, D. A. Levine, F. Dao, J. Gao, N. Schultz, C. Sander, M. Ladanyi, M. Einstein, R. Teeter, S. Benz, N. Wentzensen, I. Felau, J. C. Zenklusen, C. Bodelon, J. A. Demchok, L. Yang, M. Sheth, M. L. Ferguson, R. Tarnuzzer, H. Yang, M. Schiffman, J. Zhang, Z. Wang, T. Davidsen, O. Olaniyan, C. M. Hutter, H. J. Sofia, D. A. Gordenin, K. Chan, S. A. Roberts, L. J. Klimczak, C. Van Waes, Z. Chen, A. D. Saleh, H. Cheng, J. Parfitt, J. Bartlett, M. Albert, A. Arnaout, H. Sekhon, S. Gilbert, M. Peto, J. Myers, J. Harr, J. Eckman, J. Bergsten, K. Tucker, L. A. Zach, B. Y. Karlan, J. Lester, S. Orsulic, Q. Sun, R. Naresh, T. Pihl, Y. Wan, H. Zaren, J. Sapp, J. Miller, P. Drwiega, A. I. Ojesina, B. A. Murray, H. Zhang, A. D. Cherniack, C. Sougnez, C. S. Pedamallu, L. Lichtenstein, M. Meyerson, M. S. Noble, D. I. Heiman, D. Voet, G. Getz, G. Saksena, J. Kim, J. Shih, J. Cho, M. S. Lawrence, N. Gehlenborg, P. Lin, R. Beroukhim, S. Frazer, S. B. Gabriel, S. E. Schumacher, K. M. Leraas, T. M. Lichtenberg, E. Zmuda, J. Bowen, J. Frick, J. M. Gastier-Foster, L. Wise, M. Gerken, N. C. Ramirez, L. Danilova, L. Cope, S. B. Baylin, H. B. Salvesen, C. P. Vellano, Z. Ju, L. Diao, H. Zhao, Z. Chong, M. C. Ryan, E. Martinez-Ledesma, R. G. Verhaak, L. Averett Byers, Y. Yuan, K. Chen, S. Ling, G. B. Mills, Y. Lu, R. Akbani, S. Seth, H. Liang, J. Wang, L. Han, J. N. Weinstein, C. A. Bristow, W. Zhang, H. S. Mahadeshwar, H. Sun, J. Tang, J. Zhang, X. Song, A. Protopopov, K. R. M. Shaw, L. Chin, O. Olabode, A. I. Ojesina, P. DiSaia, A. Radenbaugh, D. Haussler, J. Zhu, J. Stuart, P. Chalise, D. Koestler, B. L. Fridley, A. K. Godwin, R. Madan, G. Ciriello, C. Martinez, K. Higgins, T. Bocklage, J. T. Auman, C. M. Perou, D. Tan, J. S. Parker, K. A. Hoadley, M. D. Wilkerson, P. A. Mieczkowski, T. Skelly, U. Veluvolu, N. The Cancer Genome Atlas Research, M. Albert Einstein College of, S. Analytical Biological, H. Barretos Cancer, M. Baylor College of, H. Beckman Research Institute of City of, A. Buck Institute for Research on, C. Canada's Michael Smith Genome Sciences, S. Harvard Medical, F. G. C. C. Helen, S. Research Institute at Christiana Care Health, B. HudsonAlpha Institute for, L. L. C. Ilsbio, M. Indiana University School of, V. Institute of Human, B. Institute for Systems, C. International Genomics, B. Leidos, H. Massachusetts General, U. McDonnell Genome Institute at Washington, W. Medical College of, C. Medical University of South, C. Memorial Sloan Kettering Cancer, C. Montefiore Medical, NantOmics, I. National Cancer, A. N. National Hospital, I. National Human Genome Research, S. National Institute of Environmental Health, D. National Institute on, D. Other Communication, L. H. S. C. Ontario Tumour Bank, O. I. f. C. R. Ontario Tumour Bank, T. O. H. Ontario Tumour Bank, H. Oregon, U. Science, C.-S. M. C. Samuel Oschin Comprehensive Cancer Institute, S. R. A. International, S. St Joseph's Candler Health, E. The, L. B. I. o. M. I. o. T. Edythe, U. Harvard, H. The Research Institute at Nationwide Children's, U. The Sidney Kimmel Comprehensive Cancer Center at Johns Hopkins, B. The University of, M. D. A. C. C. The University of Texas, H. University of Abuja Teaching, B. University of Alabama at, I. University of California, C. University of California Santa, C. University of Kansas Medical, L. University of, C. University of New Mexico Health Sciences, H. University of North Carolina at Chapel, Integrated genomic and molecular characterization of cervical cancer. *Nature* **2017**, *543*, 378.

[19] T. Z. Tan, Q. H. Miow, Y. Miki, T. Noda, S. Mori, R. Y. J. Huang, J. P. Thiery, Epithelial‐mesenchymal transition spectrum quantification and its efficacy in deciphering survival and drug responses of cancer patients. *EMBO Mol. Med.* **2014**, *6*, 1279.

[20] M. Lang, M. Binder, J. Richter, P. Schratz, F. Pfisterer, S. Coors, Q. Au, G. Casalicchio, L. Kotthoff, B. Bischl, mlr3: A modern object-oriented machine learning framework in R. *J. Open Source Softw.* **2019**, *4*, 1903.

[21] Y. Li, B. Yang, Y. Ma, X. Peng, Z. Wang, B. Sheng, Z. Wei, Y. Cui, Z. Liu, Phosphoproteomics reveals therapeutic targets of esophageal squamous cell carcinoma. *Signal Transduct. Target. Ther.* **2021**, *6*, 381.

[22] L. Li, D. Jiang, Q. Zhang, H. Liu, F. Xu, C. Guo, Z. Qin, H. Wang, J. Feng, Y. Liu, W. Chen, X. Zhang, L. Bai, S. Tian, S. Tan, C. Xu, Q. Song, Y. Liu, Y. Zhong, T. Chen, P. Zhou, J.-Y. Zhao, Y. Hou, C. Ding, Integrative proteogenomic characterization of early esophageal cancer. *Nat. Commun.* **2023**, *14*, 1666.

[23] H. Yang, Q. Zhang, M. Xu, L. Wang, X. Chen, Y. Feng, Y. Li, X. Zhang, W. Cui, X. Jia, CCL2-CCR2 axis recruits tumor associated macrophages to induce immune evasion through PD-1 signaling in esophageal carcinogenesis. *Mol. Cancer* **2020**, *19*, 41.


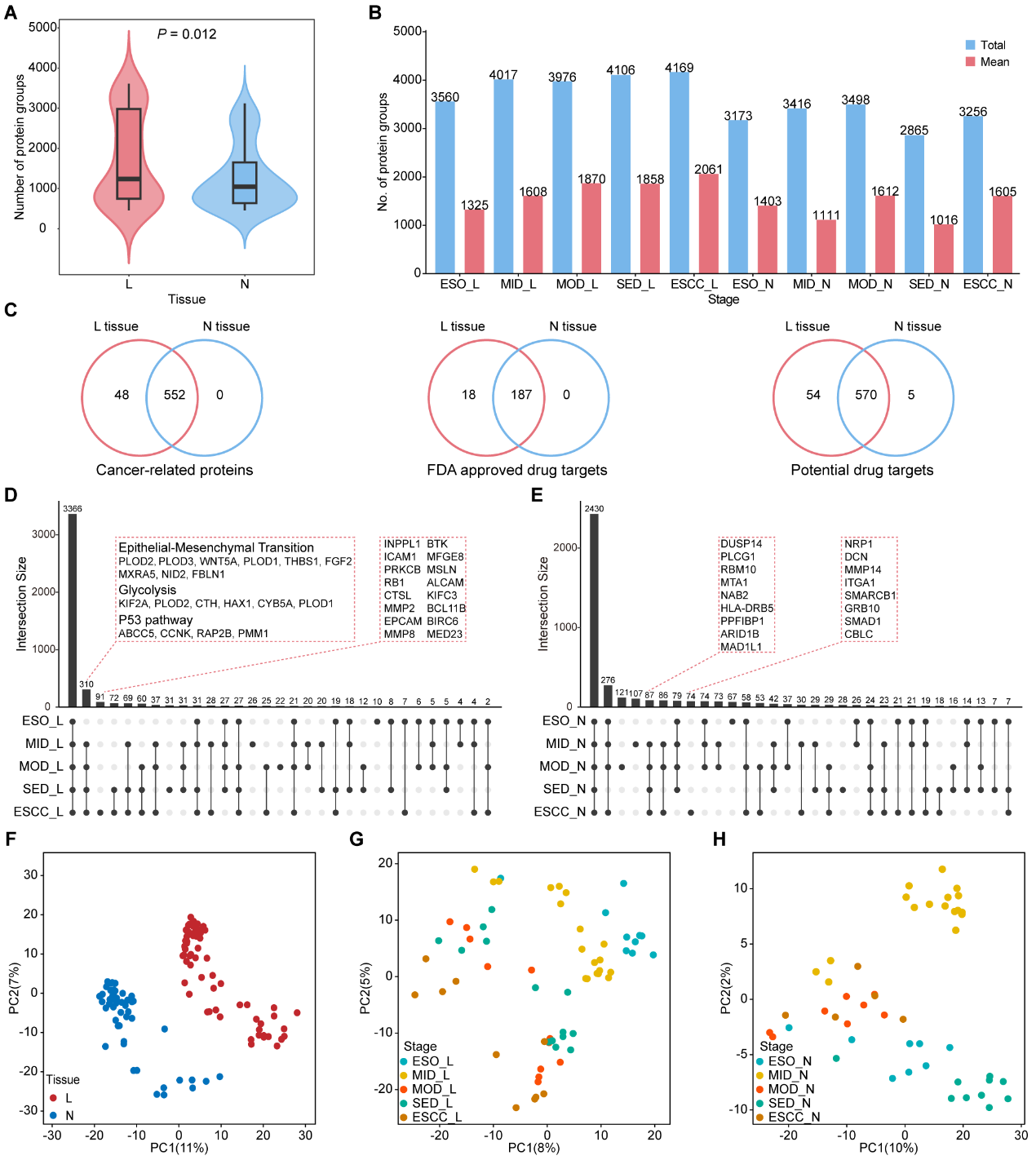


**Figure S1.** Overview of proteomic data on ESCC progression, related to Figure 2. (A) Violin plot shows the number of identified protein groups in L and N tissues. The *P* value was calculated by the two-sided unpaired Student’s t test. (B) The number of protein groups identified in L and N tissues at different stages, respectively. Total number and mean values are summarized. (C) Venn diagram shows the overlap of cancer-related proteins, FDA approved targets, and potential drug targets between L and N tissues. (D) Upset plot shows the overlap of protein groups across various stages in L tissues. The left box shows the proteins involved in the biological pathway. The right box shows the cancer-related proteins annotated by the HPA database. (E) Upset plot shows the overlap of protein groups across various stages in N tissues. The boxes show the cancer-related proteins annotated by the HPA database. (F) The Partial Least Squares Discriminant Analysis (PLSDA) of the proteome displaying the clear differences between L and N tissues. (G-H) The PLSDA of the proteomic data in L tissues (G) and N tissues (H). PC, principal component.


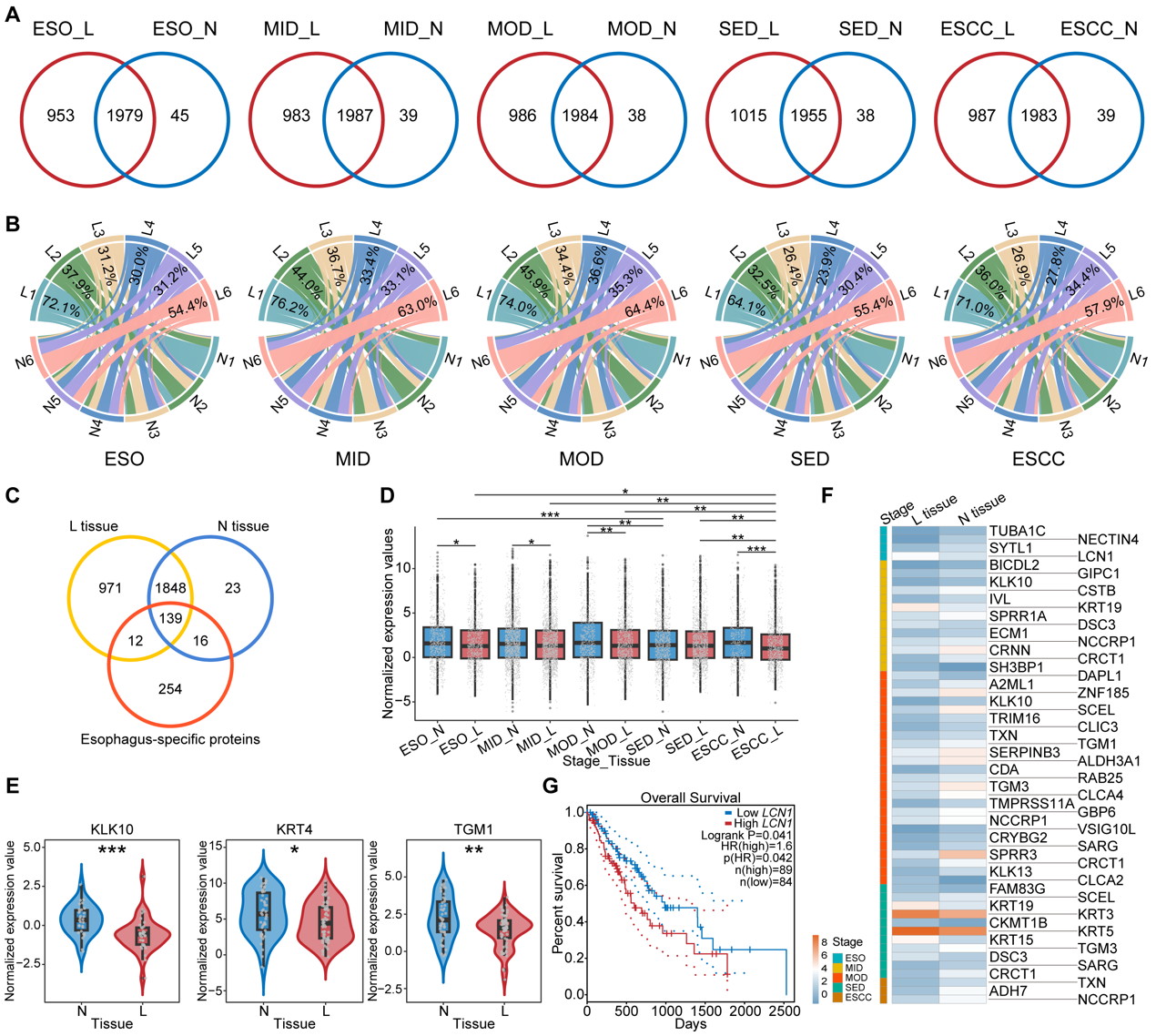


**Figure S2.** The differences in abundance patterns and esophagus-specific proteins between L and N tissues, related to Figure 2. (A) Venn diagrams show the overlap of protein groups across various stages between L and N tissues. (B) Comparison of the proteomic abundance patterns across various stages between L and N tissues. The % value indicates the proportion of the number of proteins in the same abundance rank between L and N tissues. (C) Venn diagram shows the protein overlap between esophagus-specific proteins identified in L and N tissues. (D) Boxplot shows the abundance of esophagus-specific proteins across various stages in L and N tissues. The *P* value was calculated by two-sided unpaired Wilcoxon rank-sum test (**P* < 0.05, ***P* < 0.01, ****P* < 0.001). (E) Boxplot shows the abundance of three esophagus-specific proteins in L and N tissues. The *P* value was calculated by two-sided unpaired Wilcoxon rank-sum test (**P* < 0.05, ***P* < 0.01, ****P* < 0.001). (F) The esophagus-specific proteins annotated in differentially expressed proteins across various stages between L and N tissues. (G) Kaplan-Meier survival curve comparing overall survival between patient subgroups stratified by the expression level of *LCN1* in The Cancer Genome Atlas (TCGA) cohort.

**
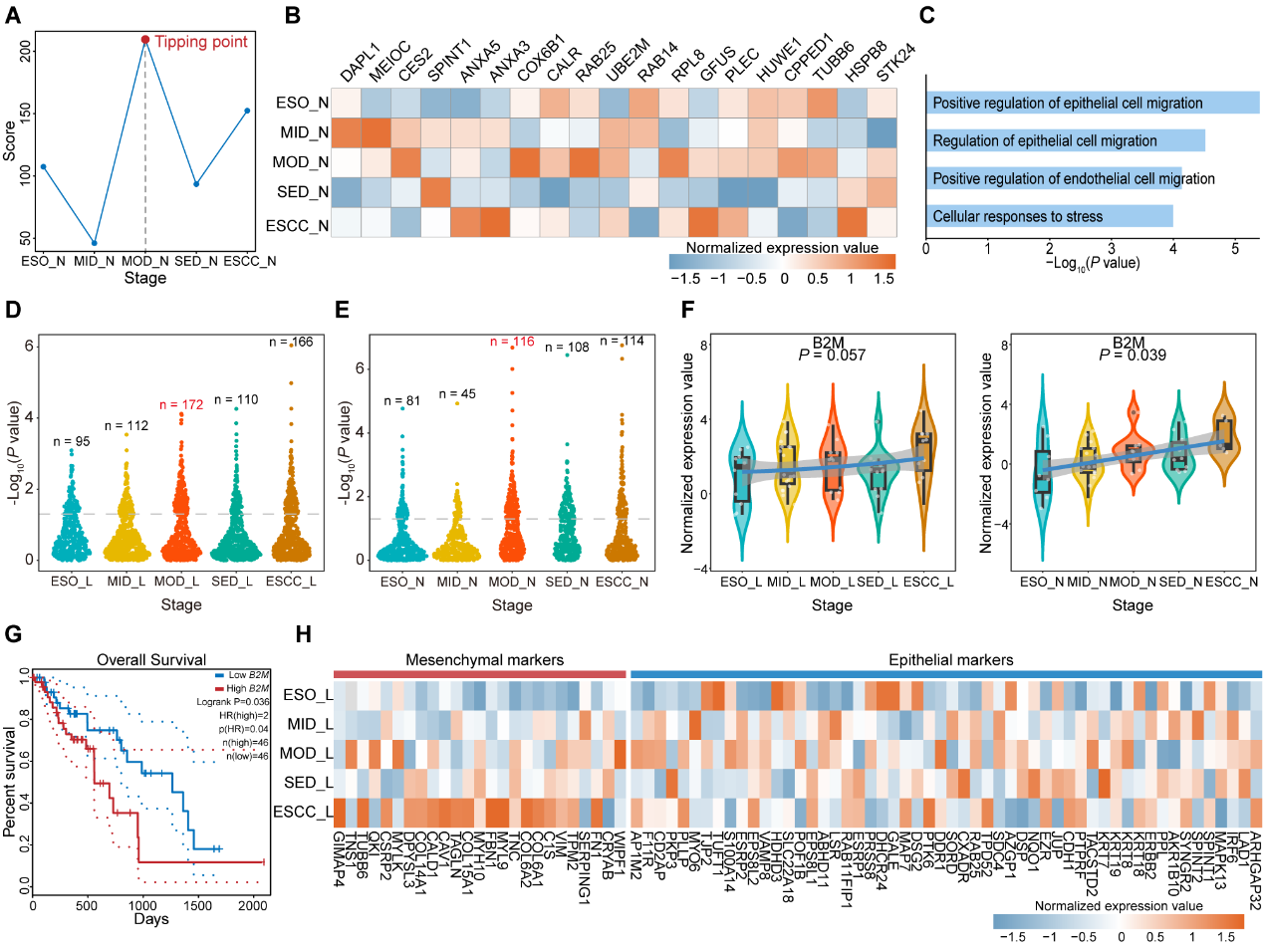
**

**Figure S3.** The MOD is a critical stage in ESCC progression and epithelial-mesenchymal transition (EMT) may play an important role in this stage, related to Figure 3. (A) The composite index (CI) scores from DNB analysis at each stage in N tissues. (B) The expression levels of DNB proteins in N tissues. (C) The enrichment analysis of DNB proteins (B) in N tissues. (D-E) The plots show the distribution of negative log10 transformed *P* values of stage-specific proteins for each stage in L (D) and N (E) tissues. The numbers on the plot indicate the count of stage-specific proteins in each stage. Stage-specific proteins were defined as differentially expressed proteins between one stage and the other four stages, with a significance threshold of *P* < 0.05 and fold change (FC) > 1.20 or FC < 0.83. (F) Violin plots show the expression level of B2M at different stages in L (left) and N (right) tissues. The *P* value was calculated by one-way analysis of variance (ANOVA). (G) Kaplan-Meier survival curve comparing overall survival between patient subgroups stratified by the expression level of *B2M* in The Cancer Genome Atlas (TCGA) cohort. (H) Heatmap shows the expression of EMT-related proteins in L tissues.

**
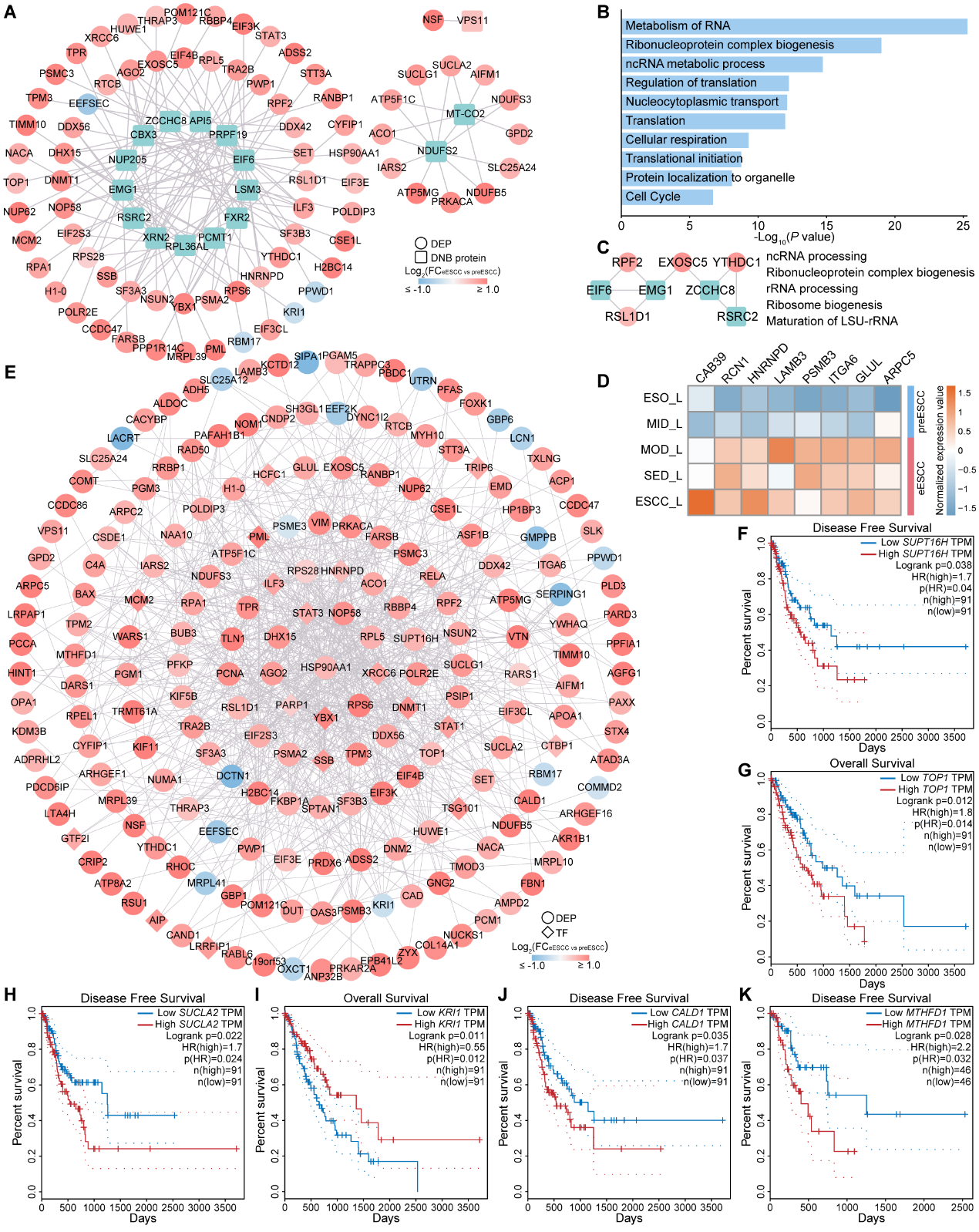
**

**Figure S4.** The proteomic differences in L tissues between preESCC and eESCC phases, related to Figure 4. (A) The protein-protein interaction (PPI) network between the dynamic network biomarker (DNB) proteins and differentially expressed proteins (DEPs) in L tissues. The squares represent DNB proteins, and the circles represent DEPs. (B) The functional enrichment analysis of proteins in (A). (C) The functional subnetwork is extracted from the protein-protein interaction (PPI) network in (A) by Molecular COmplex Detection (MCODE) in Cytoscape. (D) The expression levels of ESCC development region (EDR) proteins from Chen et al. across various stages in L tissues. (E) The PPI network of DEPs in L tissues. The circles represent DEPs and the diamonds represent the DEPs annotated as transcription factors (TFs). (F-K) Kaplan-Meier survival curve comparing prognosis between patient subgroups stratified by the expression level of *SUPT16H* (F), *TOP1* (G), *SUCLA2* (H), *KRI1* (I), *CALD1* (J), and *MTHFD1* (K) in The Cancer Genome Atlas (TCGA) cohort.


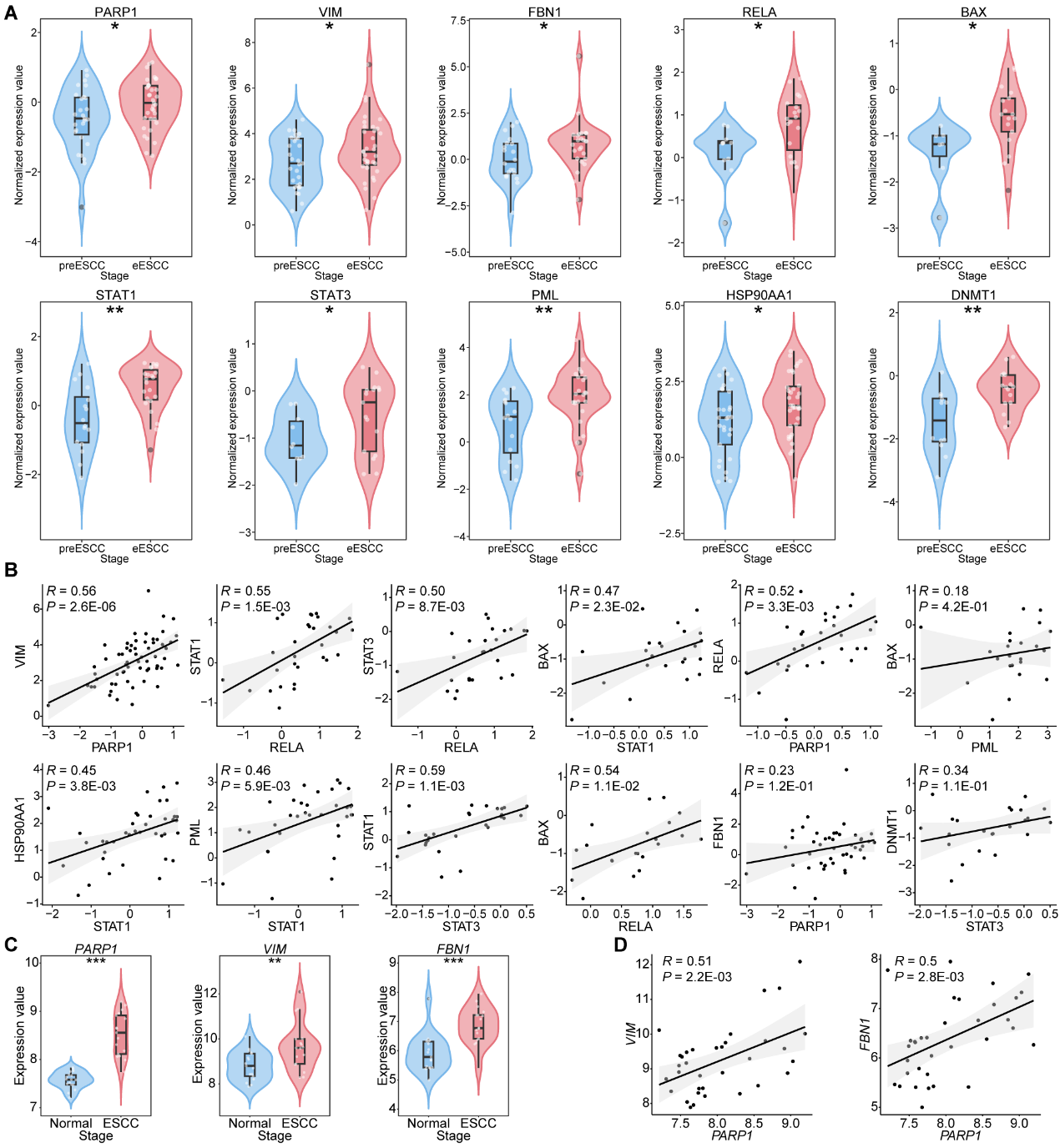


**Figure S5.** The core transcriptional regulatory network composed of ten differentially expressed proteins, related to Figure 4. (A) Violin plots show the expression levels of proteins that involved in core transcriptional regulatory network in preESCC and eESCC phases. The *P* value was calculated by Student’s t test or Wilcoxon rank-sum test, depending on whether the expression data fit the normal distribution (**P* < 0.05, ***P* < 0.01, ****P* < 0.001). (B) Pearson correlation analysis between proteins involved in composing the core transcriptional regulatory network. (C) Violin plots show the expression levels of *PARP1*, *VIM*, and *FBN1* in normal and ESCC tissues from a previous study (GSE20347). (D) Pearson correlation analysis between *PARP1* and *VIM* as well as *FBN1* in a previous study (GSE20347).

**
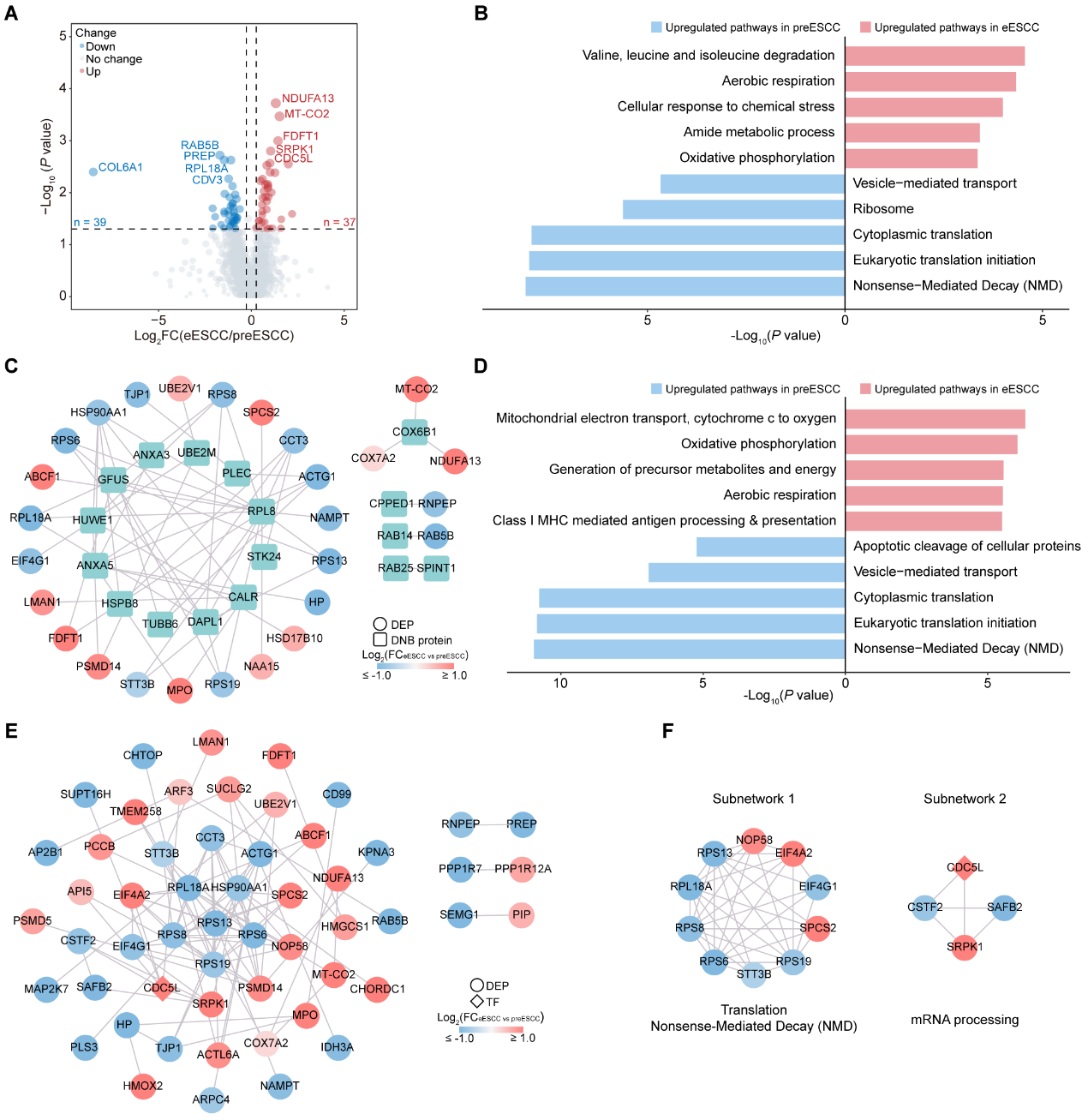
**

**Figure S6.** The proteomic differences in N tissues between preESCC and eESCC phases, related to Figure 4. (A) Volcano plot shows the differentially expressed proteins (DEPs) between preESCC and eESCC phases in N tissues. (B) The enrichment analysis of 76 DEPs (A) in N tissues. The enrichment analysis was performed using an online tool Metascape. (C) The protein-protein interaction (PPI) network between the dynamic network biomarker (DNB) proteins and differentially expressed proteins (DEPs) in N tissues. The squares represent DNB proteins, and the circles represent DEPs. (D) The functional enrichment analysis of proteins in (C). (E) The PPI network of DEPs in N tissues. The circles represent DEPs and the diamonds represent the DEPs annotated as transcription factors (TFs) by the Transcriptional Regulatory Relationships Unraveled by Sentence-based Text mining (TRRUST) database. (F) The functional subnetworks are extracted from the PPI network of DEPs by Molecular COmplex Detection (MCODE) in Cytoscape.


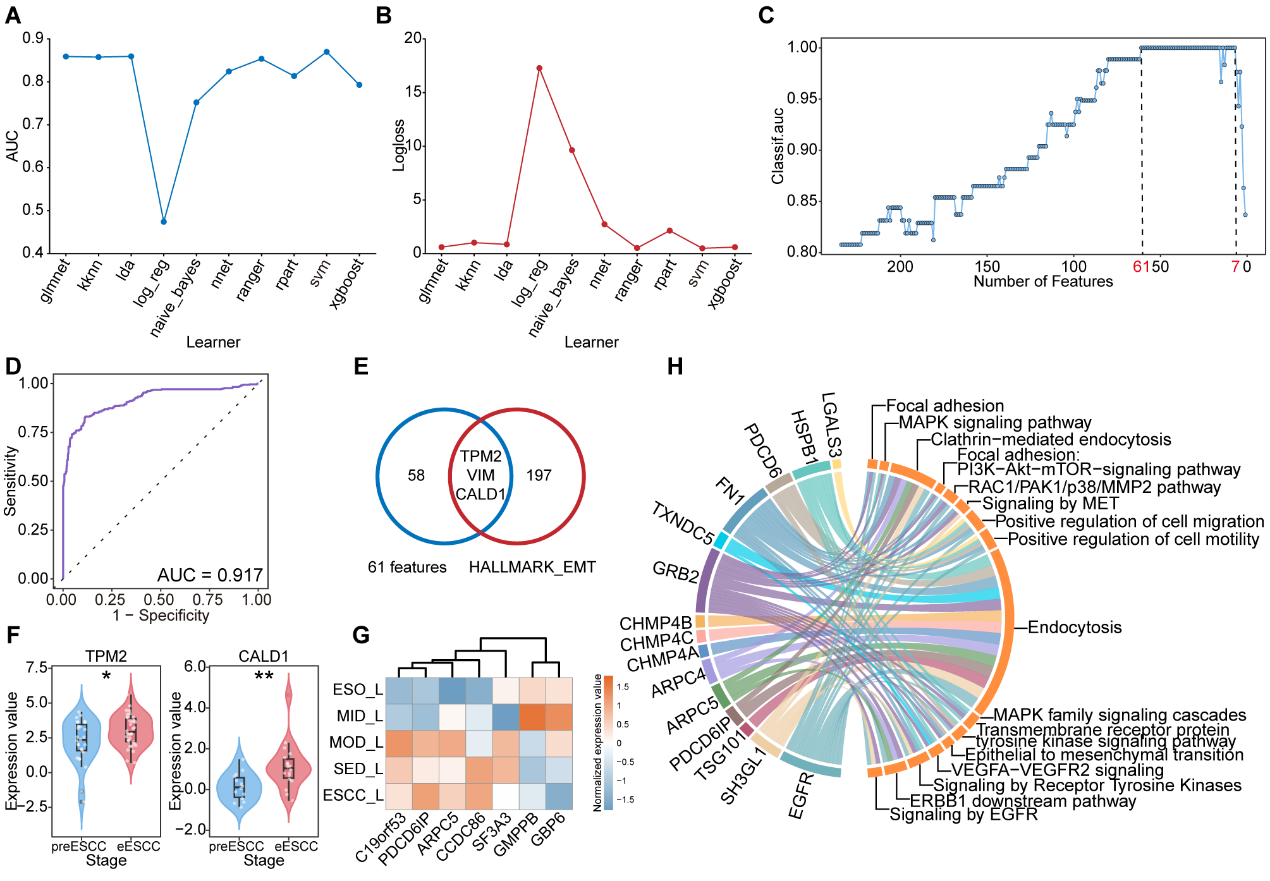


**Figure S7.** Applying machine learning to identify early biomarkers of ESCC, related to Figure 5. (A-B) The area under the curve (AUC) (A) and Logloss values (B) were obtained by benchmarking ten machine learning models. The benchmarking was performed with 10-fold cross-validation, repeated 10 times for ten machine learning models. (C) The support vector machine-recursive feature elimination (SVM-RFE) was applied to identify the most informative features for constructing machine learning model. (D) Receiver operating characteristic (ROC) curve of 61 selected features in our data. (E) Venn diagram shows the overlap between 61 selected features and the proteins involved in the epithelial-mesenchymal transition (EMT) pathway. (F) Violin plots show the expression levels of TPM2 and CALD1 in the preESCC and eESCC phases. The *P* value was calculated by Student’s t test or the Wilcoxon rank-sum test, depending on whether the expression data fit the normal distribution (**P* < 0.05, ***P* < 0.01). (G) Heatmap shows the expression of the seven most informative features in L tissues. (H) Chord diagram shows the representative biological pathways of proteins that interact with biomarkers.


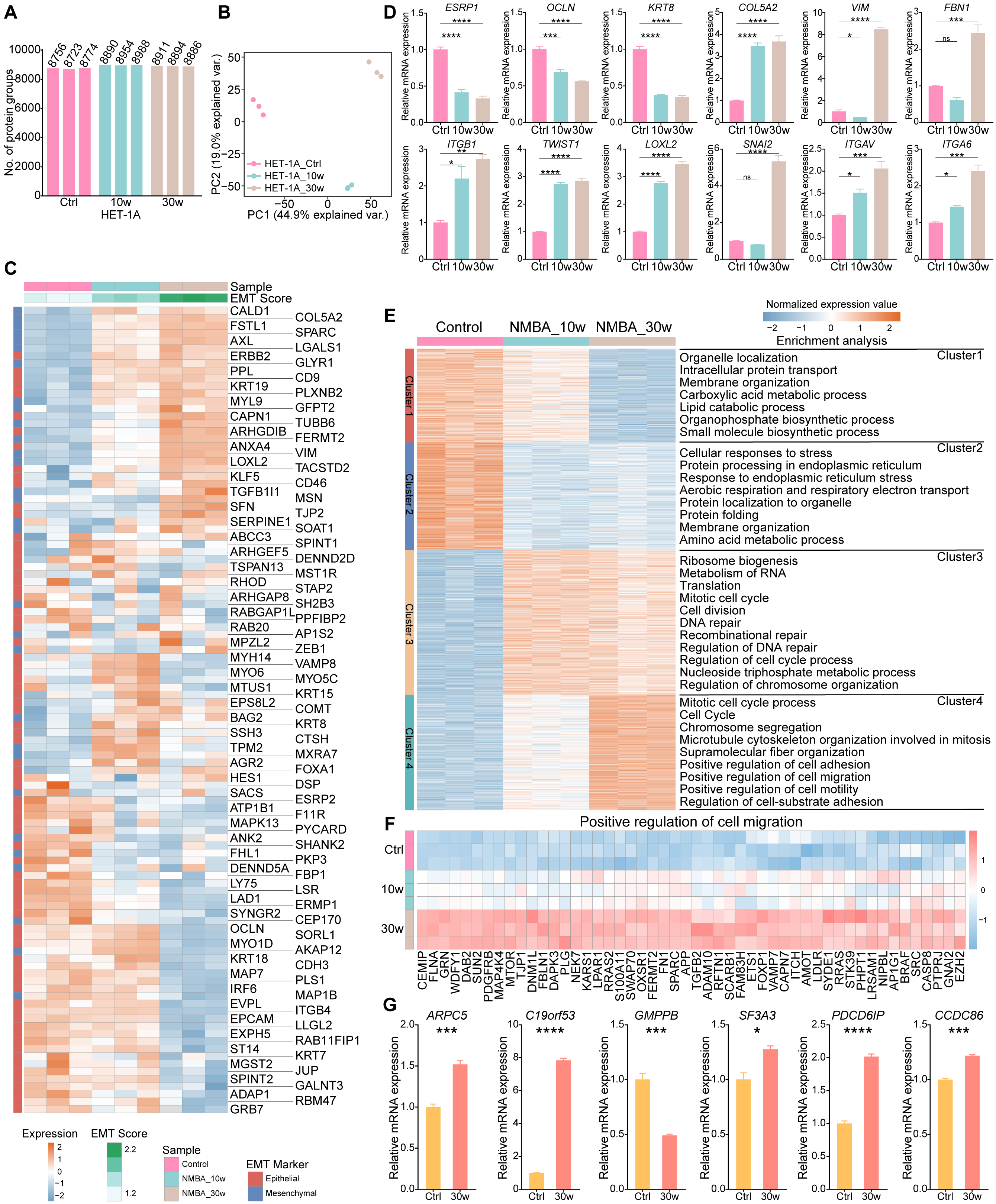


**Figure S8.** The proteomic analysis of malignantly transformed HET-1A cell model, related to Figure 6. (A) Number of quantified protein groups in HET-1A models. (B) Principal component analysis of HET-1A model proteomes. (C) Heatmap shows the expression of EMT-related proteins in malignantly transformed HET-1A cell model based on proteomic data. (D) Comparison of the mRNA expression of EMT-related genes in malignantly transformed HET-1A cell model. Data are mean ± standard error of mean (SEM) from three replications. The *P* value was calculated by Dunnett’s test. (E) The protein dynamic changes during the malignant transformation of HET-1A cell model were divided into four clusters by Mfuzz clustering. Only the proteins with a membership value greater than 0.5 were deemed core proteins of this cluster. The left panel showing the expression levels of the core proteins and the right panel showing representative biological pathways for each cluster. (F) Heatmap shows the expression of cell migration-related proteins in HET-1A model. (G) Comparison of the mRNA expression of biomarkers in control and NMBA-treated HET-1A cells (30 weeks). Data are mean ± SEM from three replications. The *P* value was calculated by Student’s t test. ns: not significant, * *P* < 0.05, ** *P* < 0.01, *** *P* < 0.001, **** *P* < 0.0001.


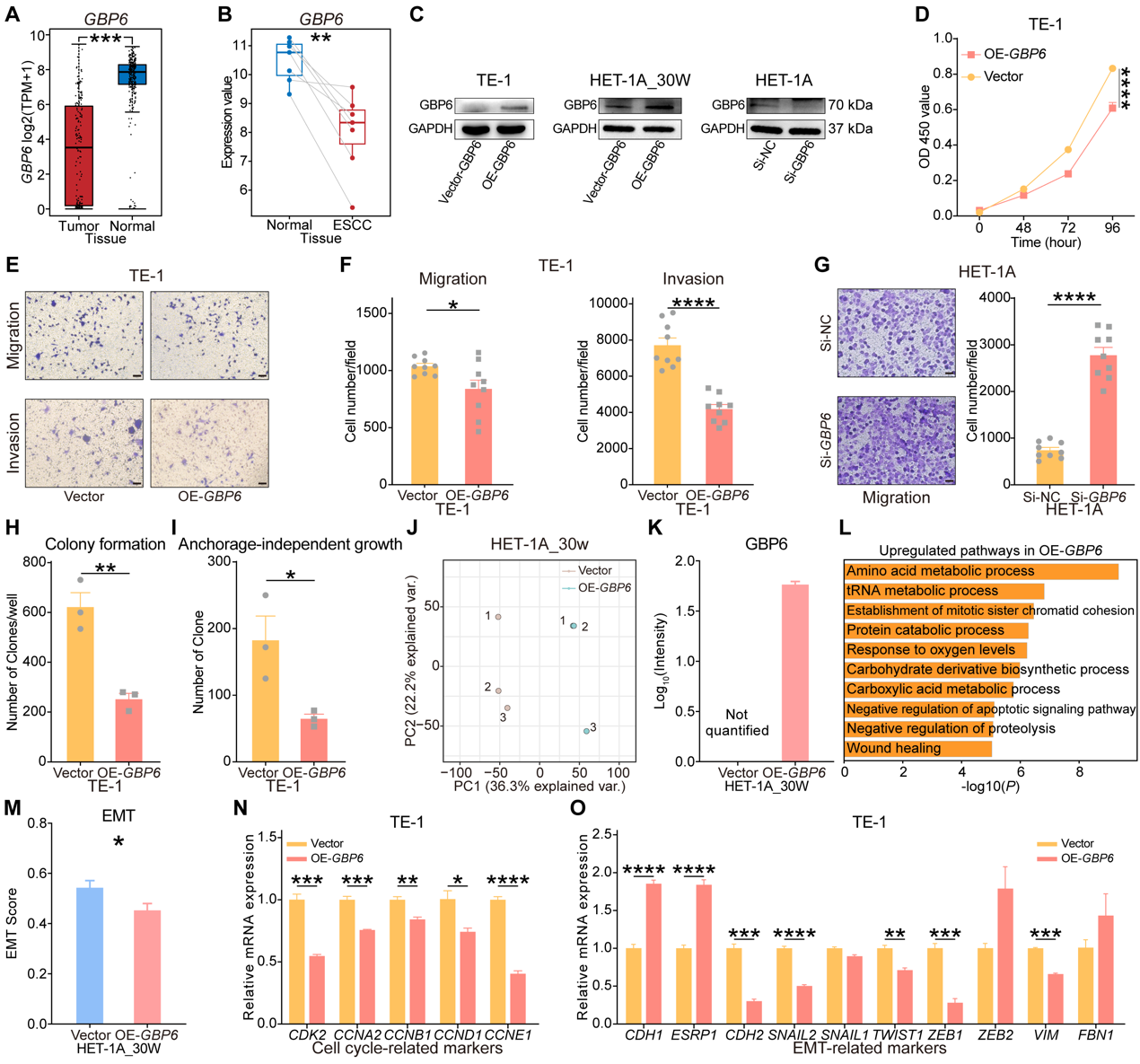


**Figure S9.** GBP6 downregulation plays an important role in promoting ESCC progression, related to Figure 6. (A) Boxplot shows the expression level of *GBP6* in The Cancer Genome Atlas (TCGA) cohort based on the Gene Expression Profiling Interactive Analysis (GEPIA) database. (B) Boxplot shows the expression level of *GBP6* in normal and ESCC tissues from a previous study (GSE77861). The *P* value was calculated by paired Student’s t test. (C) Western blotting assays of GBP6 expression level in GBP6 overexpression or knockdown TE-1, HET-1A_30w, and HET-1A cells. (D) The effects of *GBP6* overexpression on the proliferation of TE-1 cells. The *P* value was calculated by Dunnett’s test. (E-F) Representative images (E) and quantification statistics (F) show the effects of *GBP6* overexpression on TE-1 cell migration and invasion. Scale bar, 50 μm. The *P* value was calculated by Student’s t test. (G) Representative images (left) and quantification statistics (right) show the effects of *GBP6* knockdown on HET-1A cell migration. Scale bar, 50 μm. The *P* value was calculated by Student’s t test. (H-I) The effects of *GBP6* overexpression on TE-1 cell colony formation (H) and anchorage-independent growth (I). The *P* value was calculated by Student’s t test. (J) Principal component analysis of proteomics data from control and *OE-GBP6* groups in HET-1A_30w cells. (K) Proteomics data confirms that *GBP6* was overexpressed in HET-1A_30w cells. (L) The significantly upregulated pathways after GBP6 overexpression in HET-1A_30w cells. (M) Comparison of EMT scores between control and *OE-GBP6* groups in HET-1A_30w cells. The *P* value was calculated by Student’s t test. (N-O) Quantitative PCR shows the effects of *GBP6* overexpression on the altered expression of genes involved in the cell cycle (N) and EMT (O) in TE-1 cells. The *P* value was calculated by Student’s t test. * *P* < 0.05, ** *P* < 0.01, *** *P* < 0.001, **** *P* < 0.0001. OE, overexpression; EMT, epithelial-mesenchymal transition.


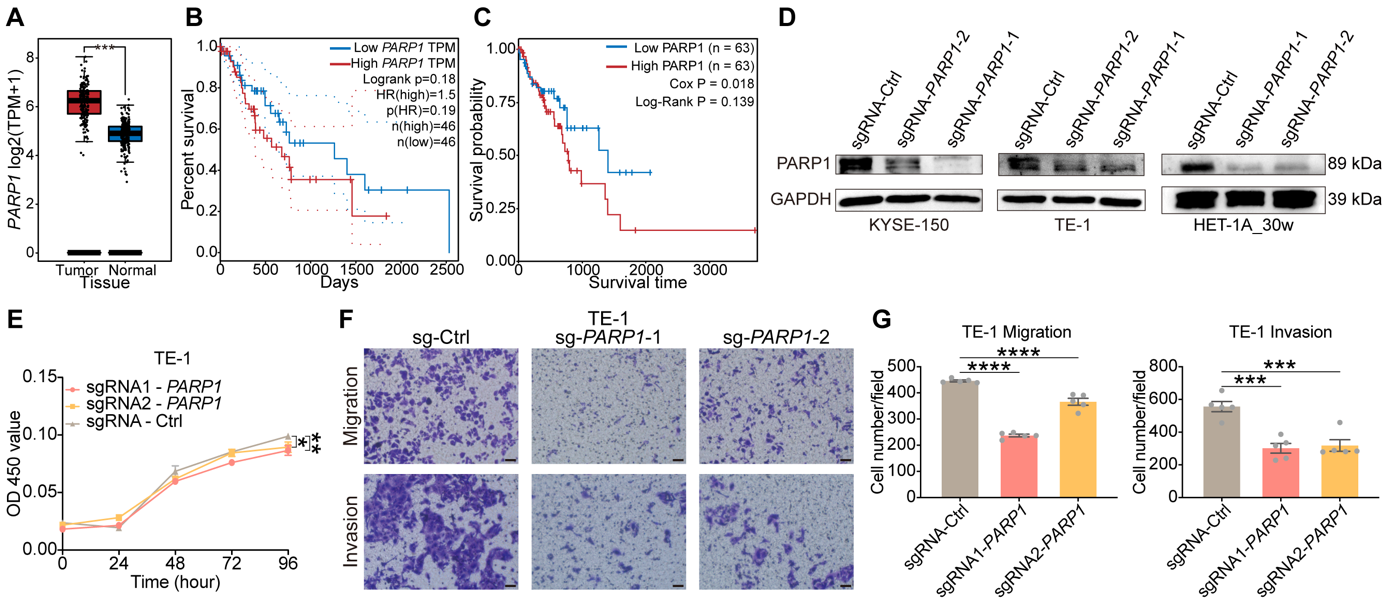


**Figure S10.** PARP1 inhibition could prevent ESCC progression, related to Figure 7. (A) Boxplot shows the expression level of *PARP1* in The Cancer Genome Atlas (TCGA) cohort based on the Gene Expression Profiling Interactive Analysis (GEPIA) database. (B) Kaplan-Meier survival curve comparing overall survival between patient subgroups stratified by the mRNA expression level of *PARP1* in TCGA cohort. Survival analysis was performed using the GEPIA database. (C) Kaplan-Meier survival curve comparing overall survival between patient subgroups stratified by the protein expression level of PARP1 in TCGA cohort. (D) Western blotting assays of PARP1 levels in *PARP1* knockout KYSE-150, TE-1, and malignantly transformed HET-1A cells. (E) The effects of *PARP1* knockout on cell proliferation of TE-1 cells. Data are mean ± SEM from five replications. The *P* value was calculated by two-way ANOVA followed by Dunnett’s test. (F-G) Representative Transwell images (F) and quantification statistics (G) show the effects of *PARP1* knockout on TE-1 cell migration and invasion. Scale bar, 50 μm. Data are mean ± SEM from five random fields. The *P* value was calculated by the Dunnett’s test. **P* < 0.05, ***P* < 0.01, ****P* < 0.001, *****P* < 0.0001.


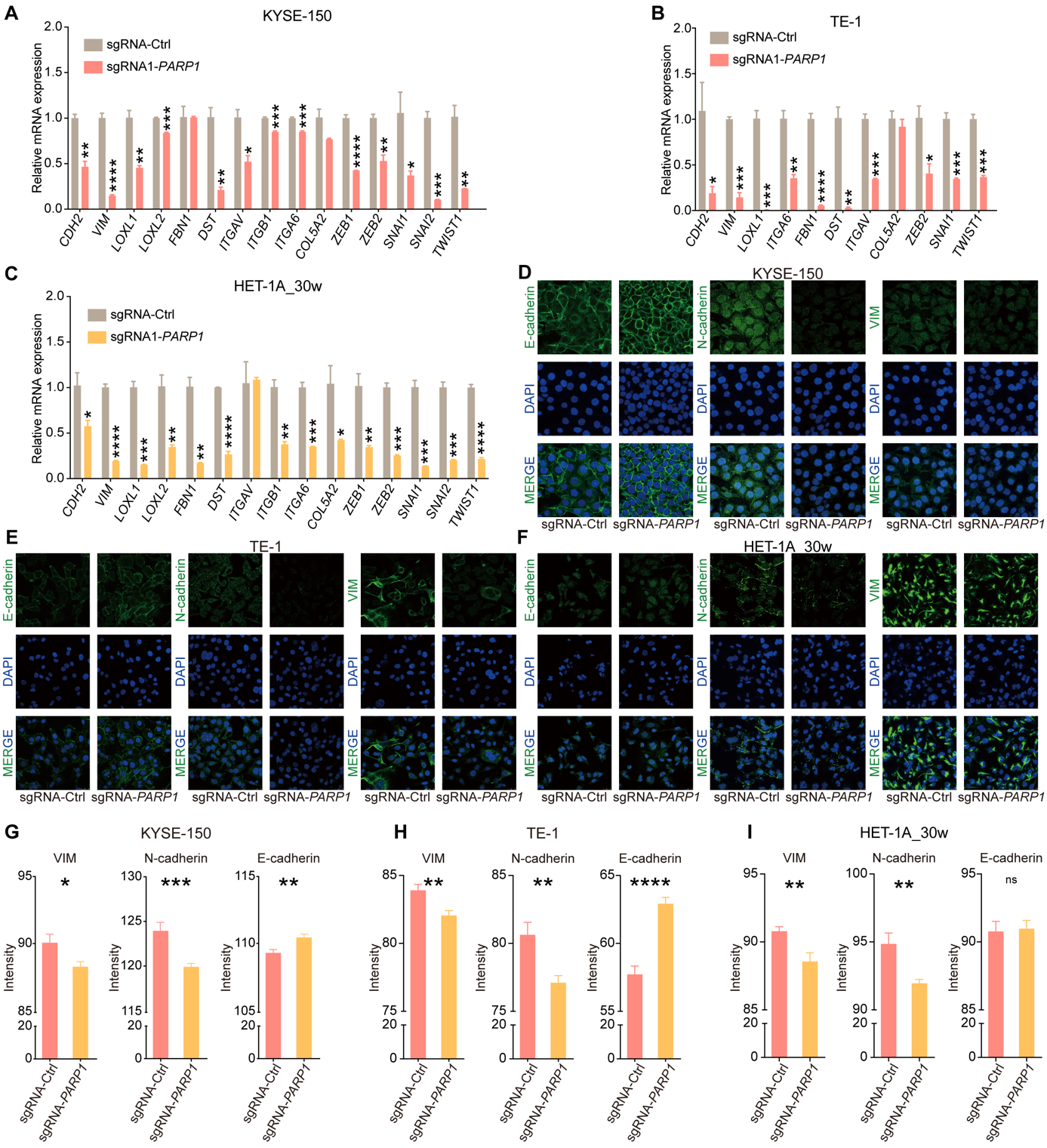


**Figure S11.** Targeting PARP1 could inhibit the EMT in ESCC progression, related to Figure 7. (A-C) Quantitative PCR shows the effects of *PARP1* knockout on the altered expression of genes involved in the EMT pathway in KYSE-150 (A), TE-1 (B), and HET-1A_30w (C) cells. Data are mean ± SEM from three replications. The *P* value was calculated by Student’s t test. (D-I) Representative images (D, E, F) and quantification statistics (G, H, I) of VIM, E-cadherin, and N-cadherin immunofluorescence staining in KYSE-150 (D, G), TE-1 (E, H), and HET-1A_30w (F, I) cells with *PARP1* knockout. Scale bar, 25 μm. Data are mean ± SEM from nine random fields. The *P* value was calculated by Student’s t test. ns: not significant, **P* < 0.05, ***P* < 0.01, ****P* < 0.001, *****P* < 0.0001.


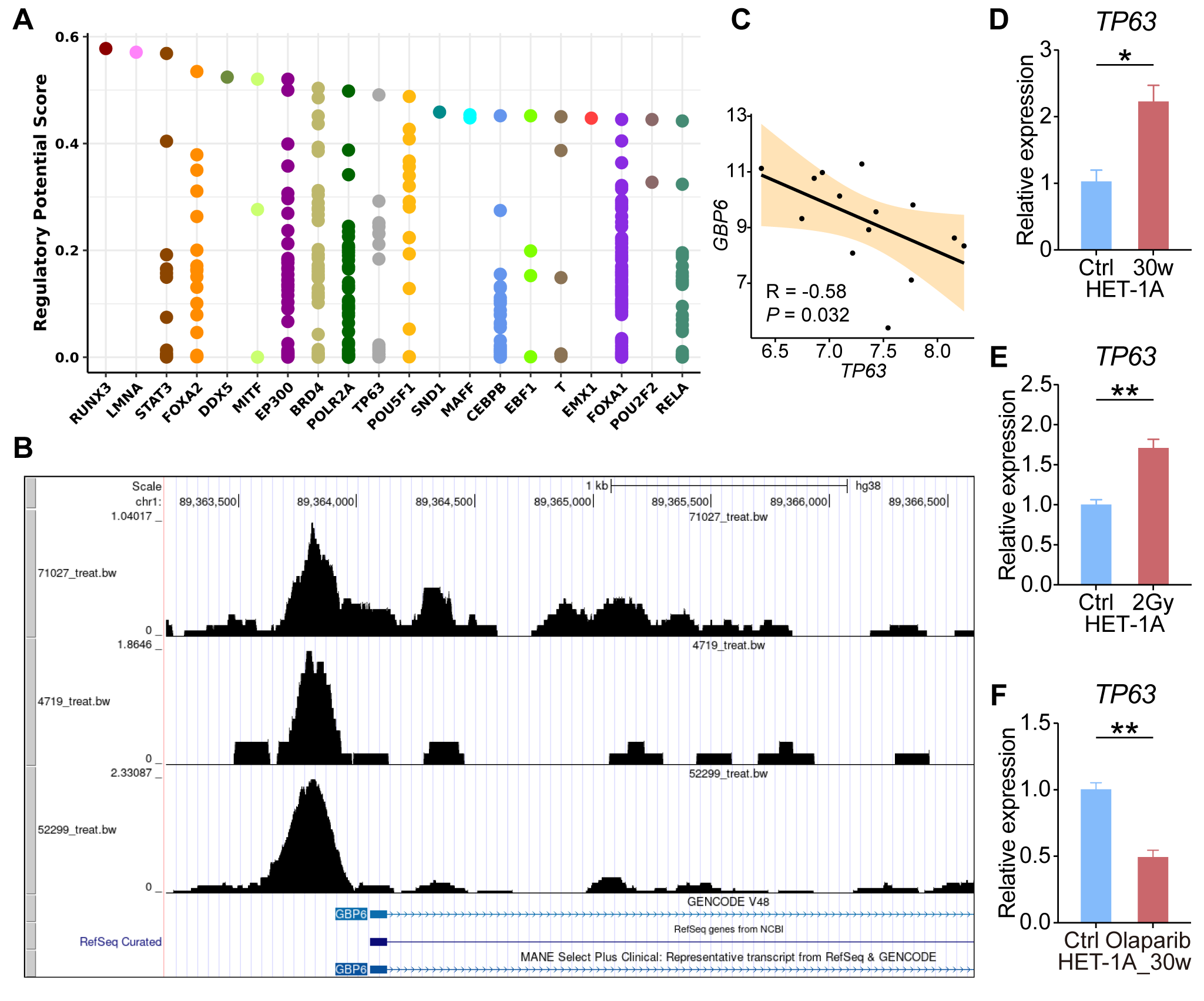


**Figure S12.** TP63 is a potential transcriptional repressor of GBP6, related to Figure 7. (A) The potential transcription factors of GBP6 obtained from the Cistrome Data Browser. (B) The Chip-seq data in Cistrome Data Browser were used to evaluate TP63 binding to the promotor region of GBP6. (C) Spearman correlation analysis between *TP63* and *GBP6* in a previous study. (D) Comparison of the mRNA expression of *TP63* in HET-1A model. (E) The mRNA expression of *TP63* was compared in HET-1A cells between the control group and the radiation exposure group. (F) Effect of PARP1 inhibitor olaparib on *TP63* expression in HET-1A_30w cells. The *P* value was calculated by Student’s t test. **P* < 0.05, ***P* < 0.01.
